# Substrate-driven microbial specialization and cooperative dechlorination of chlorinated pollutants in estuarine ecosystems

**DOI:** 10.1128/aem.00235-26

**Published:** 2026-06-02

**Authors:** Hongyan Wang, Xin Wang, Zongming Xiu, Haiwei Wei, Hongming Cai, Jiubin Chen, Tong Zhang, Yi Yang

**Affiliations:** 1 Key Laboratory of Forest Ecology and Silviculture, Institute of Applied Ecology, Chinese Academy of Sciences 74763 , Shenyang, Liaoning, China; 2 Water Resources Research Center, University of Hawaii at Manoa, Honolulu, Hawaii, USA; 3 Department of Civil, Environmental and Construction Engineering, University of Hawaii at Manoa, Honolulu, Hawaii, USA; 4 School of Environment and Geography, Qingdao University 12593, Qingdao, Shandong, China; 5 Institute of Applied Ecology, Chinese Academy of Sciences 74763 , Shenyang, Liaoning, China; 6 School of Earth System Science, Tianjin University, Tianjin, China; 7 College of Environmental Science and Engineering, Nankai University, Tianjin, China; 8 Shanghai Key Laboratory of Polar Life and Environment Sciences, Shanghai Jiao Tong University 12474, Shanghai, China; 9 Key Laboratory of Polar Ecosystem and Climate Change, Shanghai Jiao Tong University 12474, Shanghai, China; University of Delaware, Lewes, Delaware, USA

**Keywords:** *Dehalogenimonas*, *Desulfitobacterium*, 1,1,2-trichloroethane, dihaloelimination, estuary

## Abstract

Organohalide-respiring bacteria (OHRB) are globally distributed, yet their ecological roles in marine environments remain poorly understood, with few isolates characterized from these systems. Here, we describe a stable anaerobic consortium from estuarine sediments that performs sustained dechlorination of 1,1,2-trichloroethane (1,1,2-TCA) to vinyl chloride (VC) at a rate of 126.3 ± 0.9 µM d^−1^. This activity was associated with the stable co-enrichment of two key populations, *Dehalogenimonas* and *Desulfitobacterium*, which increased to dominate the community at 49.7% and 32.5%, respectively. Metagenome-assembled genomes confirmed both populations represent novel species with distinct genomic adaptations. *Dehalogenimonas* sp. strain H harbors 24 putative reductive dehalogenase genes and complete ectoine biosynthesis pathways (*ectABC*) essential for osmotolerance, while *Desulfitobacterium* sp. strain Y represents the first cultivated marine-associated member of this genus. Proteomic analysis confirmed active expression of multiple reductive dehalogenases from strain H, strongly supporting its role as the primary dechlorinator. Concurrently, physiological and genomic data suggest that strain Y is strongly co-selected under 1,1,2-TCA-amended conditions and likely occupies a crucial supportive niche. Alongside its extensive metabolic versatility that likely buffers the consortium against environmental fluctuations, its complete *de novo* corrinoid biosynthesis pathway implies a complementary role as a vitamin B_12_ provider for the extreme corrinoid-auxotrophic strain H. This study provides evidence for a stable co-enrichment consistent with nutritional niche differentiation within native microbial communities and suggests a potential cooperative interaction between novel *Dehalogenimonas* and *Desulfitobacterium* species, advancing our understanding of halogen cycling in coastal ecosystems.

**IMPORTANCE:**

Estuaries serve as critical interfaces between terrestrial and marine ecosystems, yet the microbial processes governing chlorinated pollutant fate in these vulnerable zones remain largely unexplored. Our discovery of a novel partnership between *Dehalogenimonas* and *Desulfitobacterium* species challenges the conventional understanding that *Desulfitobacterium* is restricted to terrestrial habitats. Integrative multi-omic and physiological analyses reveal that *Dehalogenimonas* strain H serves as the highly specialized primary dechlorinator, while *Desulfitobacterium* strain Y is stably co-enriched and exhibits genomic potential to sustain the consortium by providing essential corrinoid cofactors. The identification of genomic determinants underlying salt tolerance in *Dehalogenimonas*, including ectoine and mannosylglycerate biosynthesis pathways, provides mechanistic insights into OHRB adaptation to fluctuating salinity. These findings have direct implications for developing bioremediation strategies for contaminated coastal sites and highlight the importance of characterizing microbial diversity in transitional ecosystems.

## INTRODUCTION

Halogenated organic compounds (HOCs) can be of anthropogenic or natural origin. Over 8,000 naturally occurring organohalides have been identified, with marine environments serving as a major reservoir (1). Marine organisms, including algae, sponges, corals, and microorganisms, produce organohalides through substrate-specific halogenases or haloperoxidases (2–4), while abiotic halogenation occurs via photochemical reactions, volcanic activity, and Fenton-like mechanisms (5–7). Many highly halogenated natural compounds, such as polybrominated diphenyl ethers (PBDEs), have been found buried in marine sediments dating back millions of years (8), suggesting that anaerobic dehalogenation has long occurred in marine sediments as the largest anoxic environment on Earth (9–11). Accordingly, understanding the microbial processes that mediate dehalogenation in marine and coastal ecosystems is essential for elucidating global halogen cycling and pollutant fate.

Organohalide-respiring bacteria (OHRB) couple the reductive dechlorination of HOCs to energy conservation through the activity of reductive dehalogenases (RDases), serving as key agents in the detoxification of anthropogenic pollutants and contributing to global halogen cycling in anaerobic ecosystems (12). Among these, dihaloeliminating strains within the genera *Dehalobacter*, *Trichlorobacter*, *Dehalogenimonas*, and *Desulfitobacterium* (13–23) have attracted particular attention due to their ability to reductively dechlorinate vicinal dihalogenated alkanes, such as 1,1,2-trichloroethane (1,1,2-TCA), through distinct biochemical pathways (Table S1). Since the first report of anaerobic 1,1,2-TCA dechlorination by a chlorobenzoate-enriched bioreactor containing *Desulfomonile tiedjei* strain DCB-1 in 1990 (24), the bioremediation potential of these microorganisms has been increasingly recognized. However, the vast majority of characterized OHRB originate from terrestrial and freshwater sources, leaving their ecological roles in marine and coastal environments poorly understood.

Marine and coastal ecosystems, including estuaries, are subject to strong physicochemical gradients (e.g., salinity fluctuation, redox stratification, and episodic hypoxia/anoxia) that can constrain the distribution and activity of organohalide-respiring bacteria (25–28). Coastal zones also receive long-term inputs of chlorinated solvents and related industrial chemicals, and sediments can act as persistent sinks where anaerobic transformation governs natural attenuation (28, 29). Despite this environmental relevance, dihaloelimination-capable OHRB from saline environments remain underrepresented among cultivated and genome-resolved model systems. Notably, using degenerate primers targeting characterized RDase genes, diverse and phylogenetically distinct *rdhA* sequences have been detected in subsea sediments from the Pacific Ocean near Peru, Japan, Oregon (United States), and the eastern equator (30, 31), suggesting the existence of novel, uncharacterized OHRB lineages adapted to marine conditions. However, most of these organisms remain uncultured, and the extent to which dihaloeliminating OHRB have adapted to coastal and marine environments remains poorly resolved.

The genus *Dehalogenimonas* represents a crucial group of specialists in dechlorinating aliphatic alkanes, yet only two marine-linked strains have been documented, “*Candidatus* Dehalogenimonas loeffleri” strain W from the River Wuli estuary (32) and a *Dehalogenimonas* species from the Besòs River estuary (33). The situation is even more pronounced for *Desulfitobacterium*: despite being a phylogenetically diverse genus known for metabolic versatility and broad dehalogenation capabilities, it has been associated with terrestrial and freshwater habitats. Only a single isolate, *Desulfitobacterium dichloroeliminans* strain DCA1, has been shown to dechlorinate 1,1,2-TCA via dihaloelimination (19), and no *Desulfitobacterium* strains have ever been enriched or isolated from marine or estuarine environments.

To address this knowledge gap, we investigated the organohalide-respiring potential of microbial communities inhabiting estuarine sediments from the River Wuli estuary in the Bohai Sea, a historically contaminated coastal zone. Through anaerobic enrichment with 1,1,2-TCA as the terminal electron acceptor, we established a stable, sediment-free consortium dominated by phylogenetically distinct populations of *Dehalogenimonas* and *Desulfitobacterium*. We characterized the dechlorination activity, population dynamics, and genomic features of these organisms to elucidate their ecological roles, evolutionary adaptations, and contributions to halogen cycling in marine-influenced environments. By integrating multi-omic and physiological analyses, our findings suggest a potential cooperative relationship driven by nutritional niche differentiation between an estuarine-adapted *Dehalogenimonas* and, for the first time, a marine-associated *Desulfitobacterium*. Specifically, we demonstrate that both populations exhibit strict growth dependency on 1,1,2-TCA-amended conditions, and their complementary genomic profiles support a testable hypothesis of unidirectional cross-feeding for essential corrinoid cofactors. This mechanistic understanding expands our knowledge of OHRB diversity and symbiotic interactions in coastal ecosystems.

## MATERIALS AND METHODS

### Chemicals

1,1,2-Trichloroethane (1,1,2-TCA, ≥97%) was purchased from Sigma-Aldrich (St. Louis, MO, USA). Vinyl chloride (≥99.5%) was purchased from Dalian Special Gases Co., Ltd (Dalian, Liaoning, China). Vitamin B_12_ (≥98%) was purchased from Sigma-Aldrich Chemicals (St. Louis, MO, USA). All other chemicals supplied by Macklin Co., Ltd. (Shanghai, China) or Sigma-Aldrich were of analytical grade or higher.

### Microcosms and enrichment cultures

Sampling sites were selected at the estuary where the River Wuli meets the Bohai Sea (40°44′44″N, 120°59′11″E) in Huludao City, Liaoning Province, China, which has a well-documented history of chlor-alkali production impacts (34). For all cultivation, unless otherwise noted, 120-mL glass serum bottles were used, each containing 80 mL of bicarbonate-buffered (30 mM) basal mineral salt medium (containing 1.0 g L^−1^ [0.1%, wt/vol] NaCl) chemically reduced with 0.2 mM Na_2_S, 0.2 mM L-cysteine, and 0.5 mM sodium dithiothreitol (35). Triplicate microcosm bottles were inoculated with approximately 3 g of homogenized sediment inside an anaerobic chamber (Coy Laboratory, Ann Arbor, MI), sealed with butyl rubber stoppers under a headspace of N_2_/CO_2_ (80:20, vol/vol), and supplemented with 5 mM acetate as carbon source, 10 mL H_2_ (~413 µmol, 25°C) as the electron donor, neat 1,1,2-TCA (*ca*. 5 μL or 53.7 μmol, 0.7 mM aqueous concentration) as the electron acceptor, and the Wolin vitamin (35) mix with 50 μg L^−1^ vitamin B_12_. Sediment-free enrichment cultures were obtained by three transfers (3%, vol/vol) to fresh basal mineral salt medium amended with acetate, H_2_, and 1,1,2-TCA. This sediment-free culture was maintained through subsequent transfers under consistent conditions. Triplicate autoclaved controls (30 min, 121°C) were established in parallel at each transfer to account for any abiotic loss of 1,1,2-TCA. All cultures were incubated at 30°C in the dark without agitation.

### DNA extraction and 16S rRNA gene amplicon sequencing

To identify the population(s) responsible for the observed dechlorination activity, genomic DNA was extracted from 1-mL sediment slurry or enrichment cultures using a TIANamp Soil DNA Kit (TIANGEN Biotech, Beijing, China) following the manufacturer’s instructions. Cells were harvested via vacuum filtration onto 25-mm diameter, 0.22-µm pore size cellulose membrane filters. DNA concentrations were determined using a Qubit 3.0 fluorometer (Invitrogen, Carlsbad, CA, USA). The hypervariable V3-V4 region of the prokaryotic 16S rRNA gene was amplified using the V3-V4-F and V3-V4-R primer set (Table S2) in a 25-µL assay containing 20 ng template DNA, 200 nM of each primer, 0.5 µL TransStart Taq DNA Polymerase, 2.5 µL TransStart Buffer (TransGen Biotech, Beijing, China), 200 µM of each dNTP, and nuclease-free water. PCR conditions were initial denaturation at 94°C for 3 min; 24 cycles of denaturation at 94°C for 5 s, annealing at 57°C for 90 s, extension at 72°C for 10 s, and final extension at 72°C for 5 min. Indexed adapters were added using limited-cycle PCR. The multiplexed DNA libraries were loaded onto an Illumina MiSeq instrument according to the manufacturer’s instructions (Illumina, San Diego, CA, USA). Sequencing was performed using a 2 × 250 paired-end strategy. Raw 16S rRNA sequencing reads were analyzed using the Mothur software package following the MiSeq standard operating procedures (36). Quality-controlled sequences were uploaded to the SILVAngs server for comparative analysis with default parameters (37). Sequences were grouped into operational taxonomic units (OTUs) at a 97% similarity threshold.

### Quantitative PCR

TaqMan chemistry-based qPCR primer sets (Table S2) targeting the 16S rRNA gene of *Desulfitobacterium* (Desulf-q795F, Desulf-q910R, and probe Desulf-q870P) were designed using Primer3Plus. Primer set Dhgm-q478F, Dhgm-q536R, and probe Dhgm-q500P were employed to target the 16S rRNA genes of *Dehalogenimonas*. Each 25-µL assay contained 12.5 µL of 2 × Premix Ex Taq Master Mix (Takara Bio Inc.), 0.5 μL of each primer and probe (0.2 μM final concentration), 0.5 µL of 50× ROX Reference Dye II (Takara Bio Inc.), 2 µL of DNA template, and 8.5 µL nuclease-free water. qPCR assays were performed on a QuantStudio 3 Real-Time PCR system (Applied Biosystems, Waltham, MA, USA) with the following conditions: 95°C for 30 s, followed by 40 cycles of 5 s at 95°C and 34 s at 60°C. Calibration curves were generated using independently diluted plasmid DNA standards containing a partial 16S rRNA gene fragment derived from *Desulfitobacterium* sp. strain Y and *Dehalogenimonas lykanthroporepellens* strain BL-DC-9^T^, respectively. The partial 16S rRNA gene fragment of *Desulfitobacterium* was PCR amplified using the primer set Desulf-406F and Desulf-629R (Table S2), the obtained 225-bp amplicons were cloned into a pESI-T vector using a TOPO-TA cloning kit (Invitrogen, Carlsbad, CA, USA). The plasmids were extracted from the *E. coli* clone and used for generating qPCR standard. The qPCR assay exhibited amplification efficiencies of 101.6% for *Dehalogenimonas* (detection limit: 8.30 × 10^1^ gene copies per reaction; linear range: 8.30 × 10^2^ to 8.30 × 10^8^ gene copies per reaction) and 81.2% for *Desulfitobacterium* (detection limit: 6.10 × 10^2^ gene copies per reaction; linear range: 6.10 × 10^3^ to 6.10 × 10^9^ gene copies per reaction).

### Metagenome sequencing, assembly, and annotation

DNA for metagenomic sequencing was extracted from a 1.6-L aliquot of the 1,1,2-TCA-dechlorinating enrichment culture by centrifugation at 13,000 × *g* for 30 min at 4°C. DNA was extracted and purified using the sodium dodecyl sulfate (SDS) method (38). The harvested DNA was analyzed by agarose gel electrophoresis and quantified using a Qubit 2.0 Fluorometer (Thermo Scientific). DNA was sequenced by Novogene Bioinformatics Technology Co., Ltd. (Beijing, China) using Illumina NovaSeq 6000 technology. Paired-end sequencing with an insert size of ~400 bp and a read length of ~150 bp yielded approximately 50 million reads. A total of 70,609,962 raw sequences were filtered using RQCFilter (v38.22) to remove low-quality bases and adapters. After trimming and filtering, the resulting 35,304,981 paired-end reads were assembled into 18,553 contigs using the JGI Metagenome Assembly Pipeline (v2.1.0) (39). Metagenomic short-read profiling and taxonomic classification were performed using Kaiju (v1.7.3) (40). Metagenomic contigs were classified into a total of 7 draft genomes using Maxbin2 (v2.2.4) (41). Quality assessment of metagenome-assembled genomes (MAGs) was conducted using CheckM with default settings for completeness and contamination evaluation (42). Taxonomic affiliation was assigned using the GTDB-Tk software toolkit and the Type (Strain) Genome Server (TYGS) (43, 44). Coding gene prediction and functional annotation were performed using the NCBI Prokaryotic Genome Annotation Pipeline (PGAP) (45).

### Comparative genomic analysis

To gain insights into the unique characteristics of the enriched *Dehalogenimonas* and *Desulfitobacterium*, their genome were subjected to comparative analysis alongside other members of these genera retrieved from GenBank. Average nucleotide identity (ANIb) values among genomes were calculated based on BLAST+ search using JSpeciesWS (46). Digital DNA-DNA hybridization (dDDH) values were estimated using formula d_2_ of the genome-to-genome distance calculator GGDC (v3.0) provided by TYGS (44).

### Proteomic analysis

Biomass for proteomic analysis was harvested from the 17th-transfer enrichment culture during active 1,1,2-TCA dechlorination. Cells from 300 mL of culture were collected by centrifugation at 13,000 × *g* for 30 min at 4°C. Proteins were extracted using SDT lysis buffer (4% SDS, 100 mM Tris-HCl), reduced with TCEP/CAA, and digested with trypsin using a filter-aided sample preparation (FASP) protocol (47). The resulting peptides were desalted using C_18_ StageTips and vacuum-dried prior to analysis. Peptide separation was performed on an Easy-nLC 1200 system (Thermo Scientific) coupled to a Q-Exactive Plus mass spectrometer (Thermo Scientific). Peptides were separated on a C_18_ analytical column (75 μm × 150 mm, 3 μm) using a 60-min gradient of 2%–100% acetonitrile in 0.1% formic acid at 300 nL/min. Mass spectra were acquired in data-dependent acquisition (DDA) mode, with full MS scans (350–1,800 *m*/*z*) at a resolution of 70,000, followed by MS/MS of the top 20 precursors using higher-energy collisional dissociation (HCD, normalized collision energy 28%). Raw data were processed using MaxQuant (version 2.4.14.0) with searches performed against a custom protein sequence database derived from NCBI Prokaryotic Genome Annotation Pipeline (PGAP) annotations of the metagenome-assembled genomes of *Dehalogenimonas* sp. strain H and *Desulfitobacterium* sp. strain Y obtained in this study. Peptide and protein identifications were filtered at a 1% false discovery rate (FDR).

### Phylogenetic analysis

Neighbor-joining tree estimation on 16S rRNA gene alignments was performed using MEGA (v11.0.9) with 1,000 bootstrap replicates (48, 49). Clustal W was used for sequence alignment. The Tamura-Nei model was used for evolutionary model selection (50), and the reliability of tree branches was tested using the bootstrap method. The resulting tree was exported as a Newick tree and uploaded to the Interactive Tree of Life (iTOL) server for visualization (https://itol.embl.de) (51). Amino acid sequences of putative RDases encoded by *Dehalogenimonas* and *Desulfitobacterium* were compared with known RDases from diverse OHRB listed in Table S3 (https://rdasedb.biozone.utoronto.ca) (52). Phylogenetic trees were constructed using the neighbor-joining or maximum likelihood method in MEGA 11.0.9 with 100 bootstraps and the Jones-Taylor-Thornton substitution model (53).

### Analytical methods

1,1,2-TCA and its products were monitored periodically on an Agilent 7890B gas chromatograph (GC) equipped with a flame ionization detector and a DB-624 capillary column as described previously (14, 48, 54). Concentrations were determined by normalizing peak areas to standard curves generated by adding known amounts of chlorinated solvents into vessels with the same total volume and gas-to-liquid ratios. The analytical method employed has a detection limit of 2.5 μM and a quantification limit of 0.07 mM for both 1,1,2-TCA and its products in the liquid phase.

## RESULTS

### Reductive dechlorination of 1,1,2-TCA under anoxic condition

**Fig 1:**
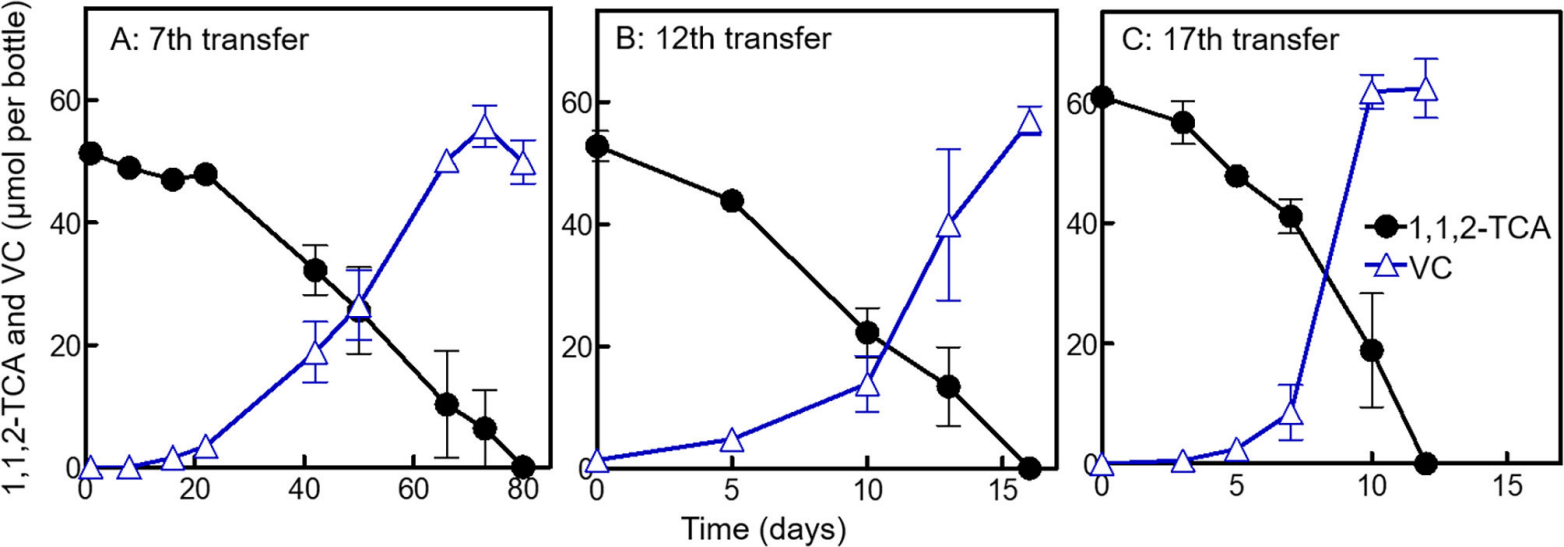
Reductive dechlorination of 1,1,2-TCA in the 7th (**A**), 12th (**B**), and 17th (**C**) transfer enrichment cultures. The initial temporal offset between substrate depletion and product accumulation is attributed to VC production below the quantification limit coupled with transient partitioning dynamics during sampling. Data points represent the means ± standard deviations of triplicate cultures.

Microcosms established with estuarine sediments from the River Wuli estuary exhibited reductive dechlorination of 1,1,2-TCA to VC within three months (data not shown) with H_2_ as the electron donor and acetate as the carbon source. Transformation activity was maintained over consecutive transfers in sediment-free enrichment cultures. In the 7th-transfer enrichment cultures, the initial 51.3 ± 1.8 µmol of 1,1,2-TCA completely disappeared with concomitant formation of 49.9 ± 3.6 µmol of VC over an 80-day incubation period, demonstrating stoichiometric microbial dechlorination of 1,1,2-TCA to VC (Fig. 1A). In the 12th-transfer enrichment cultures, 52.8 ± 2.5 µmol of 1,1,2-TCA was completely dechlorinated to VC (Fig. 1B) at a rate of 82.5 ± 3.8 µM d^−1^, representing a 5.3-fold increase compared to the 7th-transfer cultures (15.7 ± 0.6 µM d^−1^). In the 17th-transfer enrichment cultures, the dechlorination period was shortened to 12 days (Fig. 1C) with an average dechlorination rate of 126.3 ± 0.9 µM d^−1^, highlighting substantial improvement in 1,1,2-TCA dechlorination performance achieved through continuous transfers. In contrast, 1,1,2-TCA transformation was not observed in autoclaved controls. Ethene was never detected (i.e., below the detection limit) in sediment microcosms or enrichment cultures of 1,1,2-TCA, even when incubation was prolonged to 6 months or 1 year. Reductive dechlorination proceeded directly to VC without accumulation of intermediates, as 1,2-DCA or 1,1-DCA were not detected at any time point.

### Microbial community shifts reveal dehalogenimonas and desulfitobacterium dominance during 1,1,2-TCA degradation

**Fig 2:**
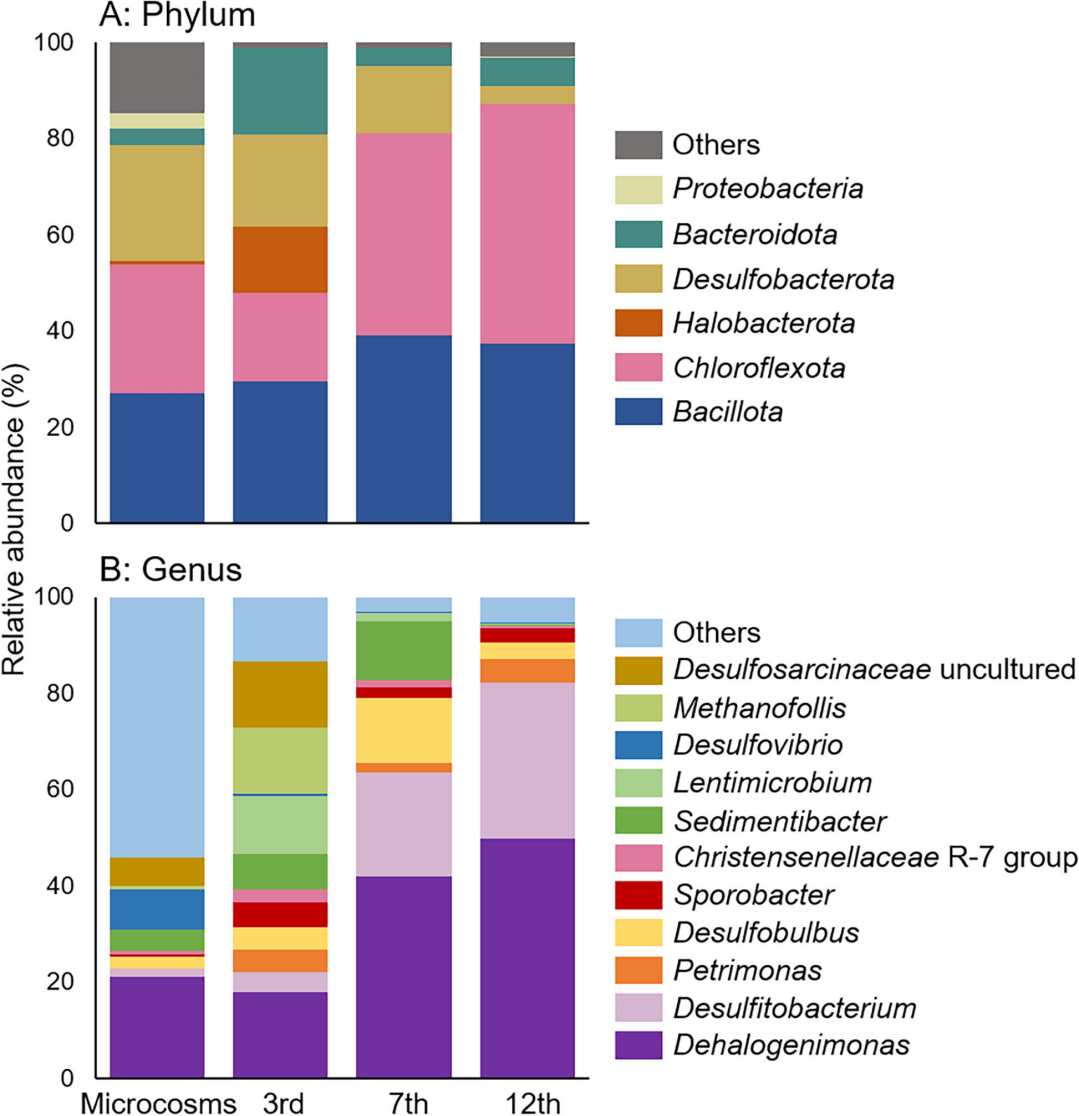
Microbial community structures of the 1,1,2-TCA-dechlorinating sediment microcosms and 3rd-, 7th-, and 12th- transfer enrichment cultures at the phylum (**A**) and genus (**B**) level.

Amplicon sequencing targeting 16S rRNA genes was performed to analyze microbial profiles in sediment microcosms and sequential enrichment cultures. A total of 85.3% of sequences obtained from sediment microcosms were classified into six major phyla: *Bacillota* (27.2%), *Chloroflexota* (26.6%), *Desulfobacterota* (23.9%), *Bacteroidota* (3.4%), *Proteobacteria* (3.2%), and *Halobacterota* (0.8%) (Fig. 2A; Table S4). Time-series analysis demonstrated dynamic shifts in community structure during transfers. By the 12th transfer, *Bacillota* and *Chloroflexota* became dominant, representing 37.5% and 49.8% of sequences, respectively. In contrast, *Halobacterota* declined from 13.7% in the 3rd transfer to below detection limits by the 12th transfer. The phylum *Desulfobacterota*, initially abundant in sediment microcosms (23.9%), decreased to 3.7% in enrichment cultures.

At the genus level (Fig. 2B; Table S5), *Desulfovibrio* (8.4%) and *Dehalogenimonas* (21.2%) dominated sediment microcosms. However, *Desulfovibrio* abundance dropped below 1% with successive transfers, while *Dehalogenimonas* increased to 49.7% by the 12th transfer. Notably, *Desulfitobacterium*, a reported OHRB within *Bacillota* with dihaloelimination capability, increased from 1.6% in sediment microcosms to 32.5% in late-stage enrichments. Archaeal sequences affiliated with *Methanofollis* decreased from 0.8% in sediment microcosms to undetectable levels in enrichments. Given prior evidence of dihaloeliminating capability of *Dehalogenimonas* (15–17) and *Desulfitobacterium* (19), coupled with their progressive enrichment in our system, we hypothesized that *Dehalogenimonas* and/or *Desulfitobacterium* species could be responsible for 1,1,2-TCA conversion.

### Growth of *Dehalogenimonas* and *Desulfitobacterium* coupled with 1,1,2-TCA reductive dechlorination

Given the 16S amplicon sequencing results, we reanalyzed DNA samples collected throughout the 3% growth-transfer experiment using *Dehalogenimonas*- and *Desulfitobacterium*-specific primers (Table S2). Following complete consumption of the initial 52.8 ± 2.5 µmol 1,1,2-TCA, *Dehalogenimonas* and *Desulfitobacterium* cell density increased 87.9- and 55.8-fold from initial values of 5.0 ± 1.2 × 10^5^ and 1.1 ± 0.1 × 10^6^ to final values of 4.4 ± 1.5 × 10^7^ and 6.2 ± 0.1 × 10^7^ cells mL^−1^ (Table S6) by the end of the 12th incubation period, respectively. Even with continued transfer to the 17th generation, *Dehalogenimonas* and *Desulfitobacterium* cell density increased 38.9- and 39.4-fold, respectively. The qPCR results demonstrated that both *Dehalogenimonas* and *Desulfitobacterium* coupled their cell growth with the reductive dechlorination of 1,1,2-TCA, whereas no growth was observed in the absence of 1,1,2-TCA (Table S6).

### Identification of the 1,1,2-TCA-dechlorinating *Dehalogenimonas* and *Desulfitobacterium* species

**Fig 3:**
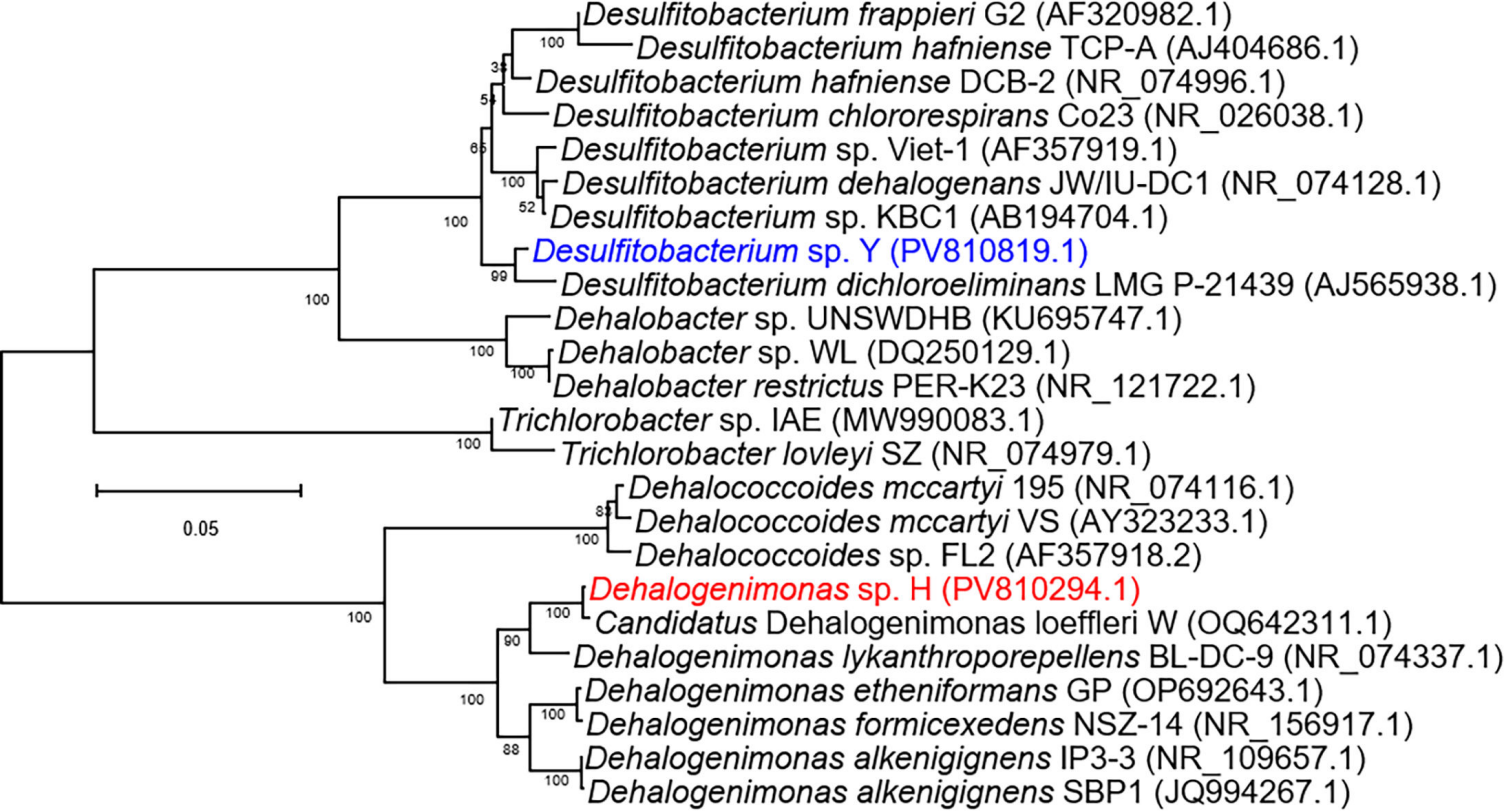
Phylogenetic tree of *Dehalogenimonas* and *Desulfitobacterium* species based on 16S rRNA gene sequences. Strain H (1,499 bp, highlighted in red) and strain Y (1,467 bp, highlighted in blue) were enriched from an estuarine environment and is compared to known *Dehalogenimonas* and *Desulfitobacterium* strains. Numbers adjacent to branches represent bootstrap support values based on 1,000 replicates. The scale bar indicates evolutionary distance as the number of nucleotide substitutions per site.

Metagenomic assembly yielded seven bins (Table S7), including one classified as *Dehalogenimonas* (96.2% completeness, 0% contamination) designated strain H, and one as *Desulfitobacterium* (98.5% completeness, 2.1% contamination) designated strain Y. Near-full-length 16S rRNA sequences of *Dehalogenimonas* sp. strain H (1,499 bp) and *Desulfitobacterium* sp. strain Y (1,467 bp) were annotated from the assembly. Phylogenetic analysis confirmed that strain H clusters within the *Dehalogenimonas* genus and strain Y belongs to *Desulfitobacterium* (Fig. 3).

To date, only one *Desulfitobacterium* strain, DCA1 (19), has been reported to dechlorinate 1,1,2-TCA to VC, sharing 98.8% sequence identity with strain Y. The second closest relative, *Desulfitobacterium hafniense* DCB-2 (97.4% identity), encodes a reductive dehalogenase targeting 3-chloro-4-hydroxyphenylacetate but lacks demonstrated activity toward 1,1,2-TCA (55). *Dehalogenimonas* sp. strain H exhibited 16S rRNA sequence identities of 99.7%, 97.6%, 96.5%, 95.8%, and 95.7% with “*Ca*. Dehalogenimonas loeffleri” strain W (18, 32), *D. lykanthroporepellens* strain BL-DC-9^T^ (15), *D. alkenigignens* strain IP3-3^T^ (56), *D. etheniformans* strain GP^T^ (57), and *D. formicexedens* strain NSZ-14^T^ (17), respectively.

### Genome annotation and analysis

The draft genome assembly of *Dehalogenimonas* sp. strain H (accession number JBOWAX000000000.1) comprises 1,944,098 bp with a G + C content of 49.5%, exhibiting distinct genome size compared to other *Dehalogenimonas* isolates (Table S8). The automated annotation pipeline identified 1,979 predicted genes, of which 1,908 were classified as protein-coding sequences and 52 as RNA genes, including 47 tRNAs and single copies of 5S, 16S, and 23S rRNA genes. Functional annotation revealed that 1,551 proteins (81.3%) could be assigned putative functions based on homology searches and conserved domain analysis, while the remaining 357 genes (18.7%) were annotated as hypothetical proteins. Pairwise comparisons among strains H, W, BL-DC-9ᵀ, GPᵀ, NSZ-14ᵀ, IP3-3ᵀ, and WBC-2 revealed ANIb values of 69.1%–80.6% and dDDH values of 16.9%–26.8% (Table S9). All comparative values fell substantially below established species demarcation thresholds (95% ANIb, 70% dDDH) (58–61), providing genomic evidence that strain H represents a novel species within the genus *Dehalogenimonas*.

**Fig 4:**
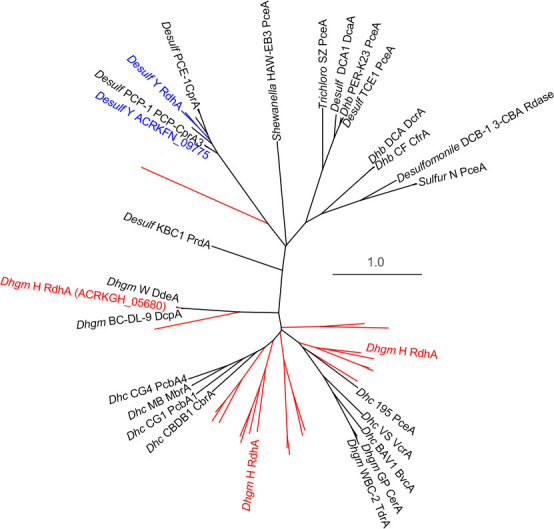
A phylogenetic tree constructed using the amino acid sequences of 44 RDases with known substrate ranges from diverse OHRB. Additionally, this tree includes the 24 putative RDase sequences from *Dehalogenimonas* sp. strain H (highlighted in red) and 3 putative RDase sequences from *Desulfitobacterium* sp. Y (highlighted in blue). The scale bar represents an estimated amino acid substitution per site, and the tree branches were generated based on 100 bootstrap iterations.

A total of 24 putative RDases were identified from the draft genome of strain H, sharing 21.7%–99.0% amino acid sequence identity with characterized RDases (Fig. 4; Table S3). One primary reductive dehalogenase subunit A (RdhA, ACRKGH_05680), comprising 487 amino acids, exhibited 99.0% amino acid identity to DdeA from “*Ca*. Dehalogenimonas loeffleri” strain W and 90.8%–95.9% identity to DcpA identified in multiple dechlorinating strains, including *Dehalococcoides mccartyi* strains RC and KS (62), *Dehalogenimonas formicexedens* strain NSZ-14^T^ (17), and *Dehalogenimonas lykanthroporepellens* strain BL-DC-9^T^ (Fig. 4) (62). Genomic analysis further revealed the presence of a complete ectoine biosynthesis gene cluster (*ectABC*), encoding enzymes (EctABC, ACRKGH_05490–05500) responsible for ectoine production. This compatible solute enables osmotic adjustment under hyperosmotic conditions (63), representing a crucial adaptation for survival in saline environments as previously documented in strain W (18). Additionally, a bifunctional mannosylglycerate synthase (MGSD, ACRKGH_02770) was identified, homologous to enzymes previously characterized in *Dehalococcoides* strains as contributing to salt tolerance (64). Mannosylglycerate represents another common compatible solute among marine microorganisms adapted to high-salinity or high-temperature environments (65). Furthermore, genomic analysis revealed that strain H is an extreme corrinoid auxotroph. It lacks the upstream *de novo* corrinoid biosynthesis machinery and retains only a fragmented salvage and remodeling pathway (Table S10), indicating its strict reliance on exogenous corrinoids for RDase function.

The draft genome of *Desulfitobacterium* sp. strain Y (accession number JBOWAY000000000.1) has a total length of 3,506,241 bp with a G + C content of 44.4% (Table S11), comparable in size to *Desulfitobacterium dichloroeliminans* DCA1^T^ (3,624,449 bp) but substantially smaller than most characterized *Desulfitobacterium* species. Notably, *D. hafniense* strains represent the upper extreme of genomic expansion within this genus, with genome sizes ranging from 5.0 to 5.7 Mbp. A total of 3,390 predicted genes were annotated in the strain Y genome, of which 3,252 were protein-coding genes alongside 89 tRNA genes, 7 copies of 5S rRNA genes, 5 copies of 16S rRNA genes (including 3 partial sequences), and 3 copies of 23S rRNA genes (one partial). Pairwise comparisons among strains Y, DCA1ᵀ, DCB-2ᵀ, 853-15Aᵀ, and JW/IU-DC1ᵀ showed ANIb values ranging from 68.7% to 87.2% and dDDH values spanning 16.9% to 33.8% (Table S12), all below established species demarcation thresholds, supporting strain Y as a novel species within the genus *Desulfitobacterium* (58–61).

Unlike the obligate organohalide-respiring *Dehalogenimonas* sp. strain H, strain Y encodes a limited repertoire of reductive dehalogenases. Genomic analysis identified only three *rdhA* genes in the strain Y genome. Despite sharing 98.8% 16S rRNA sequence identity with strain Y, *D. dichloroeliminans* strain DCA1 possesses only a single *rdhA* gene. Phylogenetic reconstruction revealed that none of strain Y’s RdhAs cluster with characterized dihaloelimination RDases, including that of DCA1, exhibiting ≤33.5% amino acid sequence identity to known dihaloelimination enzymes. Notably, one RdhA homolog (ACRKFN_09775) formed a robust clade with the chlorophenol RDase PCP-CprA3 (90.8% identity) from *D. hafniense* strain PCP-1, an enzyme that has not been reported to catalyze 1,1,2-TCA dechlorination (66).

Microbial dissimilatory sulfate reduction (DSR) is a key process in Earth’s biogeochemical sulfur cycle (67). The complete reduction of sulfate to sulfide requires the sequential activity of ATP sulfurylase (Sat), APS reductase (AprAB), and dissimilatory sulfite reductase (DsrAB) together with its physiological partner DsrC. Sulfite, the product of APS reduction, is subsequently reduced by DsrAB, leading to the production of a DsrC-trisulfide (68–70). The membrane-bound DsrMKJOP complex functions as the terminal reductase in this pathway, transferring electrons from the membrane quinone pool to reduce the DsrC-trisulfide intermediate to sulfide (71). This complex is present in most organisms that harbor *dsrAB* and *dsrC*. All described *Desulfitobacterium* spp., with the exception of *D. metallireducens* (72), are able to use sulfite as a terminal electron acceptor, while only *D. hafniense* Y51 has been reported to reduce sulfate (73). Genomic analysis of strain Y revealed the presence of sulfite reduction genes, including *dsrAB* (ACRKFN_12530-12535), *dsrMKJOP* (ACRKFN_12550-12570), and *dsrC* (ACRKFN_00245 and ACRKFN_12575), indicating the genetic capacity for sulfite respiration. However, we did not identify *sat* or *aprAB* homologs in the strain Y genome, demonstrating that strain Y lacks the known genetic machinery for utilizing sulfate as an electron acceptor. This genomic profile is consistent with a sulfite-respiring but not sulfate-respiring phenotype, typical of most *Desulfitobacterium* species (74). Beyond sulfur metabolism, strain Y genome exhibits extensive metabolic versatility for estuarine adaptation. This includes complete gene inventories for dissimilatory nitrate reduction to ammonium (DNRA; e.g., *nrfA*, ACRKFN_03830/ACRKFN_10270, *nrfH*, ACRKFN_03825), robust hydrogen cycling equipped with both [NiFe]- (e.g., ACRKFN_06895-06900) and [FeFe]-hydrogenases (e.g., ACRKFN_15965), diverse mixed-acid fermentation pathways (e.g., *pfor*, ACRKFN_00425; *ackA*, ACRKFN_09330; *ldh*, ACRKFN_13105), and a complete *de novo* menaquinone (vitamin K2) biosynthesis pathway (*men* cluster, ACRKFN_11830-11855) essential for membrane electron transport. In contrast to the auxotrophic strain H, genomic analysis confirms that strain Y possesses a complete *de novo* anaerobic corrinoid biosynthesis pathway (Table S10). This genomic feature highlights its capacity to serve as a corrinoid provider within the consortium.

### Proteomic insights into dechlorination activity

To directly link genomic potential to observed activity, shotgun proteomic analysis of the enrichment culture during active 1,1,2-TCA dechlorination was performed. A total of 573 proteins from *Dehalogenimonas* strain H and 136 proteins from *Desulfitobacterium* strain Y were identified with high confidence (FDR < 1%). Notably, nine RDases were detected from strain H. The most abundant RDase (MGI2336117.1, ACRKGH_05680) ranked as the third most highly expressed protein in the *Dehalogenimonas* proteome (MS intensity = 1.1 × 10¹⁰). Notably, while qPCR analysis of substrate-omission controls demonstrated that strain Y growth was tightly associated with 1,1,2-TCA-amended conditions (Table S10Table S6), no strain Y RDases were detected above the proteomic identification threshold despite the presence of *rdhA* genes in its genome. The *Desulfitobacterium* proteome was instead dominated by proteins involved in cell wall biosynthesis, molecular chaperones, and ABC transporters.

## DISCUSSION

### A novel estuarine OHRB partnership and its ecological significance

The capacity of OHRB to reductively dechlorinate halogenated hydrocarbons has proven instrumental in the bioremediation of contaminated soil and groundwater (75). This study reports the enrichment of a stable 1,1,2-TCA-dechlorinating consortium from estuarine sediments, revealing an unprecedented partnership between two phylogenetically distinct OHRB: *Dehalogenimonas* sp. strain H and *Desulfitobacterium* sp. strain Y. Both strains represent novel species: despite high 16S rRNA gene sequence identity with their closest relatives (>98.8%), ANIb values of <87.2% and dDDH values of <33.8% fall well below the established species demarcation thresholds. This pattern of high 16S rRNA similarity coupled with substantial genomic divergence is increasingly recognized among specialized bacteria occupying distinct ecological niches. Such discordance underscores the importance of whole-genome comparisons for accurate taxonomic assignment in OHRB (76).

The discovery of strain Y is particularly noteworthy as it represents the first documented enrichment of *Desulfitobacterium* in a marine-influenced environment. Despite extensive characterization of this genus in terrestrial and freshwater ecosystems over the past three decades (77), no isolates or enrichments have been reported from saline or estuarine habitats until now. Historically, *Desulfitobacterium* species have been predominantly associated with contaminated soils, sediments, and wastewater systems. A comprehensive review explicitly predicted that *Desulfitobacterium* would not colonize marine environments, attributing this limitation to inhibitory effects of high sulfate concentrations on their growth (77). This prediction has been largely supported by subsequent isolation efforts, with no confirmed pure cultures from saline or estuarine systems reported in major surveys of organohalide-respiring bacteria (74, 78).

Recent metagenomic studies have detected *Desulfitobacterium* sequences in marine and estuarine sediment samples, as well as in organohalide-respiring microcosms derived from these environments. However, these populations were typically transient, disappearing after successive transfers, which precluded functional and genomic characterization (79). The instability of *Desulfitobacterium* in previous marine enrichments left its ecological role and adaptive potential in saline systems largely unexplored. In contrast, our study demonstrates the stable maintenance of *Desulfitobacterium* strain Y through multiple transfers under 1,1,2-TCA selection in estuarine-derived medium, enabling comprehensive genome-resolved and proteomic characterization of this co-enriched population. This finding challenges the prevailing assumption that *Desulfitobacterium* is ecologically restricted to freshwater environments and substantially expands the known ecological niche of this genus to include transitional estuarine zones with fluctuating salinity.

### Proteomic evidence for functional engagement

The functional annotation of reductive dehalogenases (RDases) remains challenging in the study of OHRB, largely due to difficulties in heterologously expressing these membrane-associated enzymes and the often-poor correlation between sequence similarity and substrate specificity (52). Our integrated genomic and proteomic approach revealed a striking asymmetry in RDase expression between the two consortium members. Multiple RDases were detected from *Dehalogenimonas* strain H, with the DdeA-like enzyme ranking among the most abundantly expressed proteins. This protein shares high sequence identity with the characterized DdeA from “*Ca*. Dehalogenimonas loeffleri” strain W, which catalyzes dihaloelimination of 1,2-dichloroethane (18), strongly supporting strain H as the primary catalyst for 1,1,2-TCA dihaloelimination.

In contrast, no RDases from *Desulfitobacterium* strain Y were detected despite the presence of three *rdhA* genes in its genome, a relatively low number compared to some other *Desulfitobacterium* strains, which can harbor up to seven RDases, but only a few have been biochemically characterized (e.g., CprA for chlorophenols and PceA for tetrachloroethene dechlorination). Substrate-omission controls show that strain Y enrichment is contingent on 1,1,2-TCA-amended dechlorinating conditions although direct energy conservation via strain Y organohalide respiration remains to be validated. This apparent discrepancy between physiological co-enrichment and the lack of proteomic RDase detection likely reflects low protein abundance or technical limitations, such as the inherent challenges in detecting highly hydrophobic membrane-bound proteins via standard bottom-up LC-MS/MS. Alternatively, it suggests a limited direct role of strain Y RDases under the tested conditions, implying that strain Y may capitalize on the active dechlorinating environment through cooperative interactions rather than functioning as a primary competitor for the halogenated electron acceptor.

The consistent accumulation of VC as the terminal product, without further conversion to ethene, is explained by the absence of genes encoding vinyl chloride reductases in both genomes. Achieving complete dechlorination would necessitate co-culture with or bioaugmentation by VC-respiring organisms such as *Dehalococcoides mccartyi* strains harboring *vcrA* or *bvcA* (80–82), or *Dehalogenimonas etheniformans* strain GP harboring *cerA* (83, 84).

### Genomic reconstruction of the cooperative mechanism

**Fig 5:**
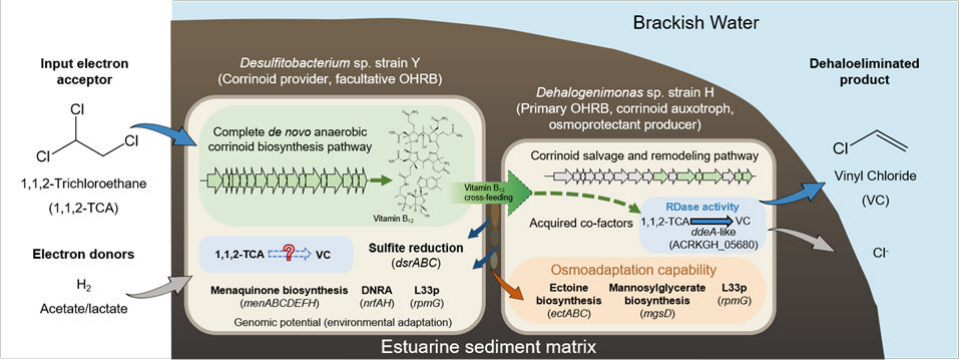
Conceptual model of the cooperative 1,1,2-trichloroethane (1,1,2-TCA) dechlorination in estuarine sediments. The diagram illustrates the genomic- and proteomic-based interactions between *Dehalogenimona*s sp. strain H and *Desulfitobacterium* sp. strain Y. Strain H functions as the primary dechlorinator, coupling 1,1,2-TCA reduction to vinyl chloride (VC). Strain Y is hypothesized to support this process by providing *de novo* synthesized corrinoids (vitamin B_12_) to the auxotrophic strain H. The model also depicts their distinct environmental adaptations: osmoregulation in strain H and extensive alternative respiration pathways (e.g., DNRA, sulfite reduction, and menaquinone biosynthesis) in strain Y. Solid arrows represent evidence-supported pathways (e.g., proteome-detected RDases), whereas dashed arrows indicate hypothesized interactions (e.g., B_12_ cross-feeding and potential dechlorination by strain Y).

We propose a genomic-based cooperative model driven by functional and nutritional niche differentiation (Fig. 5). Functionally, the proteomic and physiological data establish strain H as the highly specialized, primary dechlorinator, driven by abundantly expressed RDases (e.g., the DdeA-like enzyme). In parallel, strain Y is robustly co-enriched under 1,1,2-TCA selection, occupying a potentially crucial supportive niche. We hypothesize that this supportive role is primarily governed by corrinoid (vitamin B_12_) cross-feeding. This hypothesis is supported by the well-documented capacity of *Desulfitobacterium* species to synthesize corrinoids (85, 86), which are essential cofactors for the RDase function of the primary dechlorinator. Specifically, strain Y harbors a complete *de novo* biosynthesis pathway encompassing the essential *cbi* and *cob* gene clusters. In stark contrast, strain H is an extreme corrinoid auxotroph that relies solely on a fragmented salvage and remodeling machinery. This distinct distribution of corrinoid metabolism genes establishes a critical nutritional interdependency, rendering the primary dechlorinator (strain H) nutritionally dependent on its facultative partner (strain Y).

Beyond nutritional interdependency, the extensive metabolic versatility of strain Y provides a robust mechanistic explanation for its ecological fitness and stable co-dominance in the fluctuating estuarine environment. While its organohalide respiration may be auxiliary under the tested conditions, strain Y possesses complete gene inventories for DNRA, diverse mixed-acid fermentation pathways, and dissimilatory sulfite reduction (*dsrABC*). Furthermore, its complete *de novo* menaquinone (vitamin K2) biosynthesis pathway provides the essential electron transport infrastructure to sustain these diverse metabolic modes. This profound metabolic flexibility likely allows strain Y to thrive amidst the dynamic redox gradients of estuarine sediments. The parallel enrichment and ultimate co-dominance of both populations (38.9-fold vs 39.4-fold increase), despite asymmetric RDase expression, strongly aligns with a model of stable co-association and cooperative interaction rather than strict competition for the halogenated electron acceptor. These findings sharpen hypotheses regarding supportive interactions in estuarine OHRB consortia and provide a robust mechanistic foundation for future studies.

### Ecological distribution and osmoadaptive strategies

The identification of this dechlorinating activity confirms estuaries as significant OHRB reservoirs, where dynamic salinity and redox gradients act as key drivers structuring microbial communities (78, 87–90). Mounting evidence supports widespread dehalogenation of diverse halogenated compounds in marine and estuarine sediments (10, 28, 88–95). Phylogenetic analyses reveal that these saline dehalogenating communities are predominantly driven by sulfate-reducing bacteria affiliated with *Desulfovibrio* (9, 94, 96–98), as well as members of the *Chloroflexota* phylum (99). Specifically, marine-adapted *Chloroflexota* isolates highlight the critical role of this phylum in coastal halogen cycling. Prominent examples include the PCB-dechlorinating *Dehalobium chlorocoercia* strain DF-1 (95, 100), the multi-halogen-respiring *Dehalococcoides mccartyi* strain MB (93, 101), and the highly halotolerant “*Ca*. Dehalogenimonas loeffleri” strain W (18, 32). The prevalence of these groups strongly contrasts with the historical absence of *Desulfitobacterium* in saline habitats. Although metabolically versatile and well-studied in terrestrial and freshwater ecosystems (77), *Desulfitobacterium* spp. have appeared ecologically excluded from marine settings, making the co-enrichment of strain Y alongside the estuarine *Dehalogenimonas* strain H particularly noteworthy.

Our genomic analysis provides a mechanistic explanation for their estuarine adaptation through the lens of osmoregulation. Strain H harbors a complete ectoine biosynthesis pathway (*ectABC*), a compatible solute critical for halotolerance in *Dehalogenimonas* (18, 102). The presence of ectoine biosynthesis genes appears to strongly correlate with salt tolerance among *Dehalogenimonas* strains: those encoding this pathway tolerate elevated NaCl concentrations, whereas strains lacking *ectABC* exhibit impaired dechlorination under saline conditions (15–17, 56, 103). Additionally, the strain H genome comprises *mgsD*, which encodes a bifunctional mannosylglycerate synthase responsible for producing mannosylglycerate, another compatible solute associated with marine osmoadaptation. This enzyme possesses two domains homologous to mannosyl-3-phosphoglycerate synthase (MPGS) and mannosyl-3phosphoglycerate phosphatase (MPGP), which catalyze the consecutive synthesis and dephosphorylation steps to yield mannosylglycerate (64). Given that MGSD has been experimentally demonstrated to confer salt tolerance in *Dehalococcoides* strains (64), its presence in strain H strongly suggests it serves an analogous osmoprotective function *in vivo*. Recent studies demonstrate that *Dehalogenimonas* exhibits superior halotolerance compared to *Dehalococcoides*, linked to enhanced osmoregulatory gene diversity (104). Furthermore, consistent with the genomic signatures of halotolerant *Dehalococcoides* strains that utilize the ribosomal protein L33p and exhibit lower protein isoelectric points for salt adaptation, we identified the L33p gene in both strain H (ACRKGH_08200) and strain Y (ACRKFN_15335). The encoded proteins share 67.3% and 50.0% amino acid identity with that of *Dehalococcoides* strain 195 (Cornell subgroup), respectively. The co-occurrence of *ectABC*, *mgsD*, and L33p strongly suggests that strain H possesses enhanced halotolerance potential, enabling its sustained dechlorination activity under estuarine conditions.

In contrast, while strain Y harbors the L33p gene, no canonical compatible solute biosynthesis pathways (such as those for ectoine or mannosylglycerate) were identified in its genome. This lack of primary osmoprotectant synthesis may explain the historical absence of *Desulfitobacterium* from marine environments, suggesting that compatible solute biosynthesis is a key determinant for niche partitioning in saline environments. However, the persistence of strain Y in the estuarine-derived consortium, despite lacking identifiable osmoprotectant biosynthesis genes, raises intriguing questions about alternative osmoadaptive mechanisms that warrant future investigation. We emphasize that these osmoadaptive capabilities remain genomic predictions, as our enrichment cultures were maintained under standard laboratory conditions without elevated salinity. Systematic physiological characterization across a salinity gradient is, therefore, warranted to determine the actual salt tolerance of both strains and to assess the stability of their cooperative partnership under salt stress.

### Conclusions and future perspectives

This study reports the enrichment of a stable 1,1,2-TCA-dechlorinating consortium from estuarine sediments, comprising two novel species: *Dehalogenimonas* sp. strain H and *Desulfitobacterium* sp. strain Y. Integrated genomic and proteomic analyses establish strain H as the primary dechlorinator, with multiple RDases abundantly expressed during active dechlorination. Although no RDases from strain Y were detected via proteomics, physiological substrate-omission controls confirm its proliferation is strictly contingent on 1,1,2-TCA-amended conditions. Concurrently, metabolic reconstruction supports a testable hypothesis wherein strain Y occupies a crucial supportive niche, driven by its capacity to provide essential corrinoid cofactors for the auxotrophic strain H, alongside an extensive metabolic versatility (e.g., DNRA, fermentation, and sulfite reduction) that likely buffers the consortium against environmental fluctuations. Notably, strain Y represents the first documented enrichment of *Desulfitobacterium* in a marine-influenced environment, expanding the known ecological range of this genus.

The genomic signatures of osmoadaptation identified in strain H, including ectoine and mannosylglycerate biosynthesis pathways, provide mechanistic insights into OHRB adaptation to fluctuating salinity in estuarine systems. However, the *in vivo* expression and functional significance of these symbiotic and osmoadaptive pathways remain to be validated through physiological characterization and transcriptomic profiling. Future studies should also explore whether *Desulfitobacterium* can be leveraged for bioremediation in saline environments through strategies such as compatible solute supplementation or co-culture with halotolerant partners. This research advances our understanding of microbial niche partitioning in coastal ecosystems and identifies promising biological resources for the remediation of chlorinated pollutants in complex environmental settings.

## Data Availability

The raw metagenome sequences of the 1,1,2-TCA-dechlorinating culture were deposited in the Sequence Read Archive (SRA) under accession number SRR33695579. The metagenome and amplicon sequencing data have been deposited in GenBank under BioProject PRJNA1267767, with BioSample SAMN48784383 for *Dehalogenimonas* sp. H and SAMN48731113 for *Desulfitobacterium* sp. Y. The 16S rRNA gene sequence of *Dehalogenimonas* sp. H (genome accession number JBOWAX000000000.1) is available under accession number PV810294; the 16S rRNA gene sequence of *Desulfitobacterium* sp. Y (genome accession number JBOWAY000000000.1) is available under the accession number PV810819.

## SUPPLEMENTAL MATERIAL

Supplemental material aem.00235-26-s0001.docx Supplemental methods; Tables S1 to S12.

Graphical abstract aem.00235-26-s0002.tif Visual depiction of the study highlights.

ASM does not own the copyrights to Supplemental Material that may be linked to, or accessed through, an article. The authors have granted ASM a non-exclusive, world-wide license to publish the Supplemental Material files. Please contact the corresponding author directly for reuse.
