# Supplementary material for: Substrate-driven microbial specialization and cooperative dechlorination of chlorinated pollutants in estuarine ecosystems
Source: Appl Environ Microbiol. 2026 Jun 2;92(7):e00235-26. doi: 10.1128/aem.00235-26 (PMC13390484; doi:10.1128/aem.00235-26)
Supplement: Supplemental material — Supplemental methods; Tables S1 to S12. [file aem.00235-26-s0001.docx]

**Substrate-driven microbial specialization and cooperative dechlorination of chlorinated pollutants in estuarine ecosystems**

Hongyan Wang^1^, Xin Wang^2,3^, Zongming Xiu^4^, Haiwei Wei^5^, Hongming Cai^6^, Jiubin Chen^6^, Tong Zhang^7^, Yi Yang^1,8,9*^

^1^ Key Laboratory of Forest Ecology and Silviculture, Institute of Applied Ecology, Chinese Academy of Sciences, Shenyang, Liaoning, 110016, China;

^2^ Water Resources Research Center, University of Hawaii at Manoa, Honolulu, Hawaii 96822, United States;

^3^ Department of Civil, Environmental and Construction Engineering, University of Hawaii at Manoa, Honolulu, Hawaii 96822, United States;

^4^ School of Environment and Geography, Qingdao University, Qingdao, Shandong 266701, China;

^5^ Institute of Applied Ecology, Chinese Academy of Sciences, Shenyang, Liaoning, 110016, China

^6^ School of Earth System Science, Tianjin University, Tianjin, 300350, China;

^7^ College of Environmental Science and Engineering, Nankai University, Tianjin, 300350, China;

^8^ Shanghai Key Laboratory of Polar Life and Environment Sciences, Shanghai Jiao Tong University, Shanghai, 200030, China;

^9^ Key Laboratory of Polar Ecosystem and Climate Change, Shanghai Jiao Tong University, Shanghai, 200030, China.

* Corresponding author: Yi Yang, Key Laboratory of Forest Ecology and Silviculture, Institute of Applied Ecology, Chinese Academy of Sciences, 504 South Building, 72 Wenhua Road, Shenyang, Liaoning 110016, China.

Phone: +86-24-83970426, E-mail: [yangyi@iae.ac.cn](mailto:yangyi@iae.ac.cn)

Running Title: Estuarine OHRB Partnership for 1,1,2-TCA Dechlorination

KEYWORDS: *Dehalogenimonas*, *Desulfitobacterium*, 1,1,2-trichloroethane, dihaloelimination, estuary

**Supplementary Information**

Supplementary Methods

**Reductive Dechlorination of 1,1,2-TCA by Enrichment Cultures.** For all cultivation experiments, unless otherwise noted, we utilized 120 mL glass serum bottles containing 80 mL of a reduced, bicarbonate-buffered (30 mM, pH 7.2) basal mineral salt medium under a N₂/CO₂ (80/20, v/v) headspace. The basal medium contained the following (per liter): NaCl, 1.0 g; MgCl_2_•6H_2_O, 0.5 g; KH_2_PO_4_, 0.2 g; NH_4_Cl, 0.3 g; KCl, 0.3 g; CaCl_2_•2H_2_O, 15 mg; FeCl_2_•4H_2_O, 1.5 mg; CoCl_2_•6H_2_O, 190 *μ*g; MnCl_2_•4H_2_O, 100 *μ*g; ZnCl_2_, 70 *μ*g; H_3_BO_3_, 6 *μ*g; Na_2_MoO_4_•2H_2_O, 36 *μ*g; NiCl_2_•6H_2_O, 24 *μ*g; CuCl_2_•2H_2_O, 2 *μ*g; Na_2_SeO_3_•5H_2_O, 6 *μ*g; Na_2_WO_4_•2H_2_O, 8 *μ*g; NaHCO_3_, 2.52 g (30 mM); and 0.1 wt% resazurin, 0.025% (v/v). Reducing agents (e.g., 0.2 mM L-cysteine, 0.2 mM Na_2_S•9H_2_O, 0.5 mM sodium dithiothreitol) were supplemented. To support growth, the medium was supplied with the Wolin vitamin mix and an additional 50 *μ*g L^−1^ vitamin B_12_,_(1)_ 5 mM acetate as the carbon source, and 10 mL H_2_ (~413 *μ*mol, 25°C) as the electron donor. The initial electron acceptor concentrations of 1,1,2-trichloroethane (1,1,2-TCA) were 0.7 mM in the aqueous phase. Finally, each bottle was inoculated with 3% of an enrichment culture. Incubations were performed at 30°C in the dark without agitation.

**Table S1.** Selected anaerobic microorganisms capable of dechlorinating 1,1,2-trichloroethane (1,1,2-TCA), with emphasis on their environmental origins.

| OHRB | End products | Ecosystem category | Geographic location | References |
| --- | --- | --- | --- | --- |
| *Desulfomonile tiedjei* strain DCB-1 | 1,2-DCA (major) | Sewage sludge | USA | (2) |
| *Dehalogenimonas lykanthroporepellens*  strains BL-DC-8, BL-DC-9^T^ | VC | Groundwater | USA | (3) |
| *Dehalogenimonas alkenigignens*  strains IP3-3^T^, SBP-1 | VC | Groundwater | USA | (4) |
| *Dehalogenimonas formicexedens*  strain NSZ-14^T^ | VC | Groundwater | USA | (5) |
| *Desulfitobacterium dichloroeliminans*  strain DCA1^T^ | VC | Soil matrix | Italy | (6) |
| *Desulfitobacterium* sp. strain PR | 1,2-DCA, CA,  VC, Ethene (trace) | Bioreactor | Singapore | (7, 8) |
| *Dehalobacter* sp. strain WL | VC | Aquifer | USA | (9) |
| *Dehalobacter restrictus* strains CF, DCA | 1,2-DCA, VC | Aquifer | USA | (10) |
| *Dehalobacter restrictus* strain UNSWDHB | 1,2-DCA, VC | Soil | Australia | (11) |
| *Trichlorobacter* sp*.* strain IAE | VC | River sediment | China | (12) |
| *Dehalogenimonas* sp*.* strain W | VC | Estuarine sediments | China | (13) |

**Table S2.** Nucleotide sequences of the primers and probes used in this study.

| Primer name | Sequences (5’-3’) | Purpose | Target gene | References |
| --- | --- | --- | --- | --- |
| V3-V4-F | CCTACGGRRBGCASCAGKVRVGAAT^a^ | PCR | Prokaryotic 16S rRNA | (14) |
| V3-V4-R | GGACTACNVGGGTWTCTAATCC^a^ |  |  |  |
| Dsf-406F | GTACGACGAAGGCCTTCGGGT | PCR | Prokaryotic 16S rRNA | This study |
| Dsf-629R | CCCAGGGTTGAGCCCTAGGT |  |  |  |
| Dhgm16S-q478F | AGCAGCCGCGGTAATACG | qPCR | *Dehalogenimonas* 16S rRNA | (15) |
| Dhgm16S-q536R | CCACTTTACGCCCAATAAATCC |  |  |  |
| Dhgm16S-q500P | 6FAM-AGGCGAGCGTTATC-MGB^b^ |  |  |  |
| Dsf16S-q795F | TCGTGTCGTGAGATGTTGGG | qPCR | *Desulfitobacterium* 16S rRNA | This study |
| Dsf16S-q910R | CACCTTCCTCCGGTTTGTCA |  |  |  |
| Dsf16S-q870P | 6FAM-GCACTCTAGACAGACTGCCG-MGB^b^ |  |  |  |

^a^ Degenerate bases, R=A/G, B=C/G/T, S=C/G, K=G/T, V=A/C/G, N=A/C/G/T, W=A/T.

^b^ 6FAM, 6-carboxyfluorescein; MGB, minor groove binder moiety.

**Table S3.** Characterized reductive dehalogenases (RDases) used in phylogenetic analysis.

| RDases | Host OHRB | Metabolic function(s)^a^ | | GenBank accession number | References |
| --- | --- | --- | --- | --- | --- |
|  |  | Substrate(s) | Product(s) |  |  |
| DcpA | *Dhgm* BL-DC-9 | 1,2-DCP/1,2-DCA/1,1,2-TCA | Propene/Ethene/VC | WP_013218938 | (16) |
| DcpA | *Dhgm* NSZ-14 | 1,2-DCP/1,2-DCA/1,1,2-TCA | Propene/Ethene/VC | WP_083635400 | (17) |
| DdeA | *Dhgm* W | 1,2-DCP/1,2-DCA/1,1,2-TCA | Propene/Ethene/VC | WP_338739037 | (13, 18) |
| TceA | *Dhc* 195 | TCE, *c*DCE, 1,1-DCE/1,2-DCA | VC, (Ethene)^b^/ethene | AAW39060 | (19) |
| BvcA | *Dhc* BAV1 | TCE, 1,2-DCA, DCEs, VC | Ethene | AAT64888 | (20, 21) |
| DcpA | *Dhc* KS | 1,2-DCP | Propene | AGS15112 | (22) |
| DcaA | *Desulf* DCA1 | 1,2-DCA | Ethene | CAJ75430 | (23) |
| DcrA | *Dhb* DCA | 1,1-DCA | CA | AFV02209 | (21) |
| CfrA | *Dhb* CF | CF/1,1,1-TCA | DCM/1,1-DCA | AFV05253 | (21) |
| TmrA | *Dhb* UNSWDHB | CF | DCM | WP_034377773 | (19) |
| CtrA | *Desulf* PR | CF/1,1,1-TCA/1,1-DCA | DCM/CA | AGO27983 | (7) |
| CerA | *Dhgm* GP | 1,1-DCE, *c*DCE, VC | Ethene | QNT77188 | (15, 24) |
| TdrA | *Dhgm* WBC-2 | *t*DCE | VC | AKG53095 | (25) |
| PceA | *Dhc* 195 | PCE | TCE | AAW40342 | (19) |
| VcrA | *Dhc* VS | DCEs, VC | Ethene | ACZ62391 | (26, 27) |
| VcrA | *Dhc* WBC-2 | VC | Ethene | AOV99943 | (25) |
| MbrA | *Dhc* MB | PCE | *t*DCE, *c*DCE | ADF96893 | (28) |
| PceA | *Trichlorobacter* SZ | PCE, TCE | *c*DCE | ACD96581 | (29) |
| PceA | *Dhb* PER-K23 | PCE, TCE | *c*DCE | AHF10727 | (17) |
| PceA | *Desulf* PCE-1 | PCE | TCE | AAG49543 | (30) |
| PceA | *Desulf* TCE1 | PCE, TCE | *c*DCE | CAD28792 | (31) |
| PceA | *Desulf* PCE-S | PCE, TCE | *c*DCE | AAO60101 | (32) |
| PceA | *Desulf* Y51 | PCE, TCE | *c*DCE | BAE84628 | (33) |
| PrdA | *Desulf* KBC1 | PCE | TCE | BAE45338 | (34) |
| PceA | *Sulfur* DSM 12446 | PCE, TCE | *c*DCE | AHJ12791 | (35) |
| PceA | *Sulfur* N | PCE, TCE | *c*DCE | AAC60788 | (36) |
| PceA_TCE_ | *Sulfur* SL2 | PCE | TCE | AGW23615 | (37) |
| PceA_DCE_ | *Sulfur* SL2 | PCE, TCE | *c*DCE | AGW23613 | (37) |
| PceA | *Shewanella* HAW-EB3^c^ | PCE | TCE | ABV36340 | (38) |
| DebcprA | *Dhb* TCP1 | 2,4,6-TCP | 4-CP | AGC09147 | (39) |
| CprA | *Desulf* KBC1 | 2,4-DCP, 2,4,6-TCP | 4-CP | BAE45337 | (34) |
| CprA | *Desulf* Viet-1 | Ortho-chlorophenols | Chlorinated phenols | AAG49544 | (40) |
| CprA | *Desulf* PCE-1 | Ortho-polychlorinated phenols | Ortho-chlorophenols | AAG46187 | (30) |
| CprA | *Desulf* PCP-1 | PCP, TeCP, TCP | Chlorinated phenols | AAQ54585 | (41) |
| CprA | *Desulf* Co23 | Ortho-chlorophenols | Chlorinated phenols | AAL84925 | (42) |
| PCP-CprA3 | *Desulf* PCP-1 | PCP | 3-CP | AAK06764 | (41) |
| CbrA | *Dhc* CBDB1 | 1,2,3,4-TeCB, 1,2,3-TCB | DCB | CAI82345 | (43, 44) |
| PcbA1 | *Dhc* CG1 | *Meta-*, *para-*PCBs/PCE, TCE | Less chlorinated PCBs/*c*DCE, *t*DCE | AII58466 | (45) |
| PcbA4 | *Dhc* CG4 | *Meta-*, *para-*PCBs/PCE, TCE | Less chlorinated PCBs/*c*DCE, *t*DCE | WP_041340852 | (45) |
| PcbA5 | *Dhc* CG5 | *Meta-*, *para-*PCBs/PCE, TCE | Less chlorinated PCBs/*c*DCE, *t*DCE | AII60305 | (45) |
| CbdbA80 | *Dhc* CBDB1 | Brominated benzenes | Benzene | CAI82340 | (46, 47) |
| TcbA | *Dhb* TeCB1 | PCB, TCB | DCB | WP_068882928 | (19) |
| 3-CBA Rdase | *Desulfomonile* DCB-1 | 3-Chlorobenzoate | Benzoate | AFM24124 | (48) |
| Cl-HPA RDase | *Desulf* DCB-2 | Cl-HPA | HPA | WP_015942994 | (49) |

OHRB abbreviations: *Dhc*, *Dehalococcoides*; *Dhgm*, *Dehalogenimonas*; *Dhb*, *Dehalobacter*; *Desulf*, *Desulfitobacterium*; *Sulfur*, *Sulfurospirillum*

Organohalogen compound abbreviations: 1,2-DCA, 1,2-dichloroethane; 1,2-DCP, 1,2-dichloropropane; 1,1,2-TCA; 1,1,2-trichloroethane; 1,1,1-TCA, 1,1,1-trichloroethane; 1,1-DCA, 1,1-dichloroethane; CA, chloroethane; PCBs, polychlorinated biphenyls; 1,2,3,4-TeCB, 1,2,3,4-tetrachlorobenzene; 1,2,3-TCB, 1,2,3-trichlorobenzene; DCB, dichlorobenzene; CF, chloroform; DCM, dichloromethane; 1,1,1-trichloroethane; Cl-HPA, chloro-hydroxyphenylacetate; HPA, hydroxyphenylacetate; PCP, pentachlorophenol; TeCP, tetrachlorophenol; TCP, trichlorophenol; 2,4,6-TCP, 2,4,6-trichlorophenol; 2,4-DCP, 2,4-dichlorophenol, 4-CP, 4-chlorophenol, 3-CP, 3-chlorophenol.

^a^ RDase metabolic function is inferred based on expression studies in cultures that dechlorinated different chlorinated ethenes as electron acceptors and/or data generated in cell-free enzyme assays.

^b^ Ethene was reported as a co-metabolic product during TCE dechlorination.

^c^ *Shewanella sediminis* HAW-EB3 possesses *pceA* but growth via organohalide respiration has not been demonstrated.

**Table S4.** Phylum-level relative abundances of dominant microorganisms (>0.1%) based on 16S rRNA gene amplicon sequences obtained from the sediment microcosms and enrichment cultures. Sequences with relative abundance <0.1% were combined into the "Others" category.

| Phylum | Percentage of the total amplicon sequences (%) | | | |
| --- | --- | --- | --- | --- |
|  | Sediment microcosms | Enrichment cultures | | |
|  |  | 3rd | 7th | 12th |
| *Bacillota* | 27.2 | 29.7 | 39.2 | 37.5 |
| *Chloroflexota* | 26.6 | 18.3 | 42.0 | 49.8 |
| *Halobacterota* | 0.8 | 13.7 | 0.0 | 0.0 |
| *Desulfobacterota* | 23.9 | 19.2 | 13.9 | 3.7 |
| *Bacteroidota* | 3.4 | 17.9 | 3.7 | 5.9 |
| *Proteobacteria* | 3.2 | 0.0 | 0.0 | 0.2 |
| Others | 14.7 | 1.3 | 1.2 | 3.0 |

3rd, 7th and 12th represented third-, seventh- and twelfth-transfer enrichment cultures.

**Table S5.** Genus-level relative abundances of dominant microorganisms (>0.1%) based on 16S rRNA gene amplicon sequences obtained from the sediment microcosms and enrichment cultures. Sequences with relative abundance <0.1% were combined into the "Others" category.

| Genus | Percentage of the total amplicon sequences (%) | | | |
| --- | --- | --- | --- | --- |
|  | Sediment microcosms | Enrichment cultures | | |
|  |  | 3rd | 7th | 12th |
| *Dehalogenimonas* | 21.2 | 17.9 | 42.0 | 49.7 |
| *Desulfitobacterium* | 1.6 | 4.2 | 21.5 | 32.5 |
| *Petrimonas* | 0.0 | 4.6 | 1.9 | 4.9 |
| *Desulfobulbus* | 2.4 | 4.8 | 13.5 | 3.5 |
| *Sporobacter* | 0.5 | 5.0 | 2.3 | 2.8 |
| *Christensenellaceae* R-7 group | 0.6 | 2.7 | 1.5 | 0.7 |
| *Sedimentibacter* | 4.5 | 7.3 | 12.2 | 0.2 |
| *Lentimicrobium* | 0.0 | 12.1 | 1.7 | 0.2 |
| *Desulfovibrio* | 8.4 | 0.5 | 0.3 | 0.2 |
| *Methanofollis* | 0.8 | 13.7 | 0.0 | 0.0 |
| *Desulfosarcinaceae* uncultured | 5.8 | 13.8 | 0.0 | 0.0 |
| Others | 48.3 | 6.6 | 1.2 | 1.5 |

3rd, 7th and 12th represented third-, seventh- and twelfth-transfer enrichment cultures.

**Table S6.** Increase fold of *Dehalogenimonas* (*Dhgm*) and *Desulfitobacterium* (*Desulf*) in enrichment cultures amended with and without 1,1,2-TCA.

| Cultures | Host | Cell densities (cells mL^−1^) | | Fold increase |
| --- | --- | --- | --- | --- |
|  |  | initial | final |  |
| 12^th^ | *Dhgm* | 5.0 ± 1.2 × 10^5^ | 4.4 ± 1.5 × 10^7^ | 87.9 |
|  | *Desulf* | 1.1 ± 0.1 × 10^6^ | 6.2 ± 0.1 × 10^7^ | 55.8 |
| 17^th^ | *Dhgm* | 2.6 ± 0.3 × 10^6^ | 1.0 ± 0.1 × 10^8^ | 38.9 |
|  | *Desulf* | 2.0 ± 0.1 × 10^6^ | 8.0 ± 0.1 × 10^7^ | 39.4 |
| Without 1,1,2-TCA | *Dhgm* | 5.7 ± 4.3 × 10^5^ | 3.6 ± 2.6 × 10^5^ | 0.6 |
|  | *Desulf* | 4.3 ± 0.4 × 10^5^ | 2.7 ± 1.5 × 10^5^ | 0.6 |

**Table S7.** Taxonomy and relevant statistics of all seven MAGs binned from the metagenomic assembly.

| Bin Name | Classification | Completeness | Contamination | Number of Contigs | Size of MAG |
| --- | --- | --- | --- | --- | --- |
| bin.001 | d_Bacteria; p_*Chloroflexota*; c_*Dehalococcoidia*; o_*Dehalococcoidales*; f_*Dehalococcoidaceae*; g_*Dehalogenimonas* | 96.2 | 0 | 49 | 2082593 |
| bin.002 | d_Bacteria; p_*Actinobacteriota*; c_*Actinomycetia*; o_*Propionibacteriales*; f_*Propionibacteriaceae*; g_*Propionicimonas* | 97.2 | 0 | 15 | 3861043 |
| bin.003 | d_Bacteria; p_*Firmicutes_B*; c_*Desulfitobacteriia*; o_*Desulfitobacteriales*; f_*Desulfitobacteriaceae*; g_*Desulfitobacterium* | 98.5 | 2.1 | 54 | 3506241 |
| bin.004 | d_Bacteria; p_*Bacteroidota*; c_*Bacteroidia*; o_*Bacteroidales*; f_*Dysgonomonadaceae*; g_*Petrimonas* | 98.4 | 1.2 | 162 | 3328167 |
| bin.005 | d_Bacteria; p_*Bacteroidota*; c_*Bacteroidia*; o_*Bacteroidales*; f_*Lentimicrobiaceae*; g_*Lentimicrobium* | 92.6 | 4.6 | 362 | 4094586 |
| bin.006 | d_Bacteria; p_*Firmicutes_A*; c_*Clostridia*; o_*Tissierellales*; f_*Sedimentibacteraceae*; g_*Sedimentibacter*; s_*Sedimentibacter saalensis* | 99.1 | 1.8 | 52 | 3915940 |
| bin.007 | d_Bacteria; p_*Desulfobacterota_I*; c_*Desulfovibrionia*; o_*Desulfovibrionales*; f_*Desulfovibrionaceae*; g_*Desulfocurvibacter* | 79.7 | 2.9 | 1195 | 2926839 |

**Table S8.** Genome properties of selected *Dehalogenimonas* strains compiled from NCBI GenBank database.

| Organisms | Size (Mb) | G+C  (mol%) | Total genes | CDSs | tRNA | rRNA | *rdhA* | Originated from | References |
| --- | --- | --- | --- | --- | --- | --- | --- | --- | --- |
| *Dehalogenimonas* sp. H | 1.94 | 49.5 | 1,979 | 1,908 | 47 | 3 | 24 | Estuarine sediment, China | This study |
| *Candidatus* Dehalogenimonas loeffleri W | 1.77 | 52.5 | 1,822 | 1,763 | 47 | 3 | 28 | Estuarine sediment, China | (18) |
| *D. lykanthroporepellens* BL-DC-9^T^ | 1.69 | 55.0 | 1,771 | 1,720 | 47 | 3 | 25 | Contaminated groundwater, USA | (16) |
| *D. etheniformans* GP^T^ | 2.07 | 51.9 | 2,079 | 2,029 | 47 | 3 | 50 | Grape pomace compost, Germany | (15, 50) |
| *D. formicexedens* NSZ-14^T^ | 2.09 | 54.0 | 2,210 | 2,156 | 49 | 3 | 24 | Contaminated groundwater, USA | (5) |
| *D. alkenigignens* IP3-3^T^ | 1.85 | 55.9 | 1,988 | 1,936 | 47 | 3 | 29 | Contaminated groundwater, USA | (51) |
| *Dehalogenimonas* sp. WBC-2 | 1.73 | 49.2 | 1,782 | 1,730 | 46 | 3 | 22 | 1,1,2,2-TeCA-contaminated wetland sediment, USA | (25) |

**Table S9.** Average nucleotide identity (ANIb) values (%) and estimated digital DNA-DNA hybridization (dDDH) values (%) from *in silico* comparison of two *Dehalogenimonas* genome sequences. The ANIb values are presented in the lower left half of the table, while the dDDH values are shown in the upper right half.

| Strains | 1 | 2 | 3 | 4 | 5 | 6 | 7 |
| --- | --- | --- | --- | --- | --- | --- | --- |
| 1. H | - | 22.9  (20.6 - 25.4) | 18.2  (16.1 - 20.6) | 17.2  (15.0 - 19.5) | 20.0  (17.8 - 22.4) | 21.9  (19.6 - 24.3) | 17.5  (15.4 - 19.8) |
| 2. W | 80.6 | - | 19.5  (16.4 - 23.1) | 16.9  (14.8 - 19.2) | 18.1  (16.0 - 20.5) | 18.9  (16.7 - 21.2) | 17.9  (15.7 - 20.2) |
| 3. BL-DC-9^T^ | 73.1 | 73.5 | - | 18.3  (16.1 - 20.7) | 18.3  (16.1 - 20.6) | 18.7  (16.5 - 21.0) | 18.4  (16.3 - 20.8) |
| 4. GP^T^ | 69.1 | 69.6 | 70.3 | - | 21.6  (19.4 - 24.1) | 18.5  (16.3 - 20.9) | 26.8  (24.4 - 29.3) |
| 5. NSZ-14^T^ | 70.4 | 70.7 | 70.6 | 79.3 | - | 26.2  (23.9 - 28.7) | 19.1  (16.9 - 21.5) |
| 6. IP3-3^T^ | 70.7 | 71.3 | 71.2 | 72.5 | 75.8 | - | 18.6  (16.4 - 21.0) |
| 7. WBC-2 | 71.3 | 71.7 | 70.8 | 70.7 | 70.6 | 70.7 | - |

**Table S10.** Genomic inventory of corrinoid (vitamin B_12_) metabolism pathways in *Dehalogenimonas* sp. strain H and *Desulfitobacterium* sp. strain Y. Locus tags for the identified genes are provided (prefixes ACRKGH_ for strain H and ACRKFN_ for strain Y). Empty cells indicate that the corresponding gene was not identified in the genome assembly.

| Enzymes name | Gene | Strain H (ACRKGH_) | Strain Y (ACRKFN_) |
| --- | --- | --- | --- |
| uroporphyrinogen-III C-methyltransferase | *cobA* |  | 06695 |
| uroporphyrin-III C-methyltransferase / precorrin-2 dehydrogenase / sirohydrochlorin ferrochelatase | *cysG* |  | 06680 |
| sirohydrochlorin chelatase | *cbiX-like* |  | 07435 |
| precorrin-2 C (20)-methyltransferase | *cbiL* |  | 07460 |
| precorrin-3B C (17)-methyltransferase | *cbiH* |  | 07445 |
| precorrin-4 C (11)-methyltransferase | *cbiF* |  | 07455 |
| cobalt-precorrin 5A hydrolase | *cbiG* |  | 07450 |
| cobalt-precorrin-5B (C (1))-methyltransferase | *cbiD* |  | 07470 |
| precorrin-6A reductase | *cbiJ* |  | 07440 |
| precorrin-6y C5,15-methyltransferase (decarboxylating) subunit | *cbiET* | 01000 | 07465 |
| precorrin-8X methylmutase | *cbiC* |  | 07430 |
| cobyrinate a,c-diamide synthase | *cbiA* | 01155 | 07415 |
| cob(I)yrinic acid a,c-diamide adenosyltransferase | *cobA* | 01005/03490 | 07410 |
| cobyric acid synthase | *cbiP* | 02560 | 07425 |
| adenosylcobinamide-phosphate synthase | *cbiB* |  | 07420 |
| bifunctional adenosylcobinamide kinase / adenosylcobinamide-phosphate guanylyltransferase | *cobU* | 01595 | 06820 |
| adenosylcobinamide-GDP ribazoletransferase | *cobS* | 01585 | 06815 |

**Table S11.** Genome properties of selected *Desulfitobacterium* strains compiled from NCBI GenBank database.

| Organisms | Size (Mb) | G+C  (mol%) | Total genes | CDSs | 16S rRNA | *rdhA* | Halogenated electron acceptor | Originated from | References |
| --- | --- | --- | --- | --- | --- | --- | --- | --- | --- |
| *Desulfitobacterium* sp. Y | 3.51 | 44.4 | 3,390 | 3,252 | 5 | 3 | Not confirmed (co-enriched with 1,1,2-TCA) | Estuarine sediment, China | This study |
| *D. dichloroeliminans*  DCA1^T^ | 3.62 | 44.2 | 3537 | 3340 | 6 | 1 | 1,2-DCA,  1,1,2-TCA | Soil polluted with 1,2-DCA, Belgium | (6) |
| *D. hafniense* DCB-2^T^ | 5.28 | 47.5 | 5042 | 4883 | 5 | 7 | Chlorophenols | Sewage sludge, Denmark | (49) |
| *D. hafniense* Y51 | 5.73 | 47.4 | 5208 | 5060 | 6 | 2 | PCE | Soil-contaminated with PCE, Japan | (33) |
| *D. hafniense* PCP-1 | 5.56 | 47.5 | 5358 | 5243 | 3 | 7 | Chlorophenols | Methanogenic consortium from mixture of anaerobic sewage sludge and soil, Canada | (52) |
| *D. hafniense* PCE-S | 5.67 | 47.3 | 5494 | 5417 | 2 | 2 | PCE | Soil-contaminated with chlorinated ethenes, Germany | (53) |
| *D. metallireducens* 853–15A^T^ | 3.18 | 41.9 | 3163 | 3046 | 8 | 0 | - | Uranium-contaminated aquifer, USA | (54) |
| *D. dehalogenans* JW/IU-DC1^T^ | 4.32 | 45.0 | 4241 | 4011 | 6 | 6 | Chlorophenols | Freshwater pond sediment, USA | (55) |
| *D. dehalogenans* PCE1 | 4.22 | 45 | 4079 | 3977 | 3 | 6 | PCE, Chlorophenols | Chloroethene-polluted soil, The Netherlands | (52) |

**Table S12.** Average nucleotide identity (ANIb) values (%) and estimated digital DNA-DNA hybridization (dDDH) values (%) from *in silico* comparison of two *Desulfitobacterium* genome sequences. The ANIb values are presented in the lower left half of the table, while the dDDH values are shown in the upper right half.

| Strains | 1 | 2 | 3 | 4 | 5 |
| --- | --- | --- | --- | --- | --- |
| 1. Y | - | 33.8  (31.4 - 36.3) | 20.4  (18.2 - 22.8) | 19.0  (16.8 - 21.4) | 20.5  (18.3 - 22.9) |
| 2. DCA1^T^ | 87.2 | - | 19.5  (16.4 - 23.1) | 16.9  (14.8 - 19.2) | 18.1  (16.0 - 20.5) |
| 3. DCB-2^T^ | 75.6 | 75.9 | - | 18.3  (16.1 - 20.7) | 18.3  (16.1 - 20.6) |
| 4. 853–15A^T^ | 69.0 | 69.5 | 68.7 | - | 21.6  (19.4 - 24.1) |
| 5. JW/IU-DC1^T^ | 76.0 | 76.2 | 84.6 | 69.5 | - |

**References**

1. Löffler FE, Sanford RA, Ritalahti KM. 2005. Enrichment, cultivation, and detection of reductively dechlorinating bacteria. Methods Enzymol 397:77-111.

2. Fathepure BZ, Tiedje JM. 1994. Reductive dechlorination of tetrachloroethylene by a chlorobenzoate-enriched biofilm reactor. Environ Sci Technol 28:746-752.

3. Yan J, Rash BA, Rainey FA, Moe WM. 2009. Isolation of novel bacteria within the *Chloroflexi* capable of reductive dechlorination of 1,2,3-trichloropropane. Environ Microbiol 11:833-843.

4. Bowman KS, Nobre MF, da Costa MS, Rainey FA, Moe WM. 2013. *Dehalogenimonas alkenigignens* sp. nov., a chlorinated-alkane-dehalogenating bacterium isolated from groundwater. Int J Syst Evol Microbiol 63:1492-1498.

5. Key TA, Bowman KS, Lee I, Chun J, Albuquerque L, da Costa MS, Rainey FA, Moe WM. 2017. *Dehalogenimonas formicexedens* sp. nov., a chlorinated alkane-respiring bacterium isolated from contaminated groundwater. Int J Syst Evol Microbiol 67:1366-1373.

6. De Wildeman S, Diekert G, Van Langenhove H, Verstraete W. 2003. Stereoselective microbial dehalorespiration with vicinal dichlorinated alkanes. Appl Environ Microbiol 69:5643-5647.

7. Zhao S, Ding C, He J. 2015. Detoxification of 1,1,2-trichloroethane to ethene by *Desulfitobacterium* and identification of its functional reductase gene. PLoS One 10:e0119507.

8. Ding C, Zhao S, He J. 2014. A *Desulfitobacterium* sp. strain PR reductively dechlorinates both 1,1,1-trichloroethane and chloroform. Environ Microbiol 16:3387-3397.

9. Grostern A, Edwards EA. 2006. Growth of *Dehalobacter* and *Dehalococcoides* spp. during degradation of chlorinated ethanes. Appl Environ Microbiol 72:428-436.

10. Wang PH, Tang S, Nemr K, Flick R, Yan J, Mahadevan R, Yakunin AF, Löffler FE, Edwards EA. 2017. Refined experimental annotation reveals conserved corrinoid autotrophy in chloroform-respiring *Dehalobacter* isolates. ISME J 11:626-640.

11. Wong YK, Holland SI, Ertan H, Manefield M, Lee M. 2016. Isolation and characterization of *Dehalobacter* sp. strain UNSWDHB capable of chloroform and chlorinated ethane respiration. Environ Microbiol 18:3092-3105.

12. Jiang L, Yang Y, Jin H, Wang H, Swift CM, Xie Y, Schubert T, Loffler FE, Yan J. 2022. *Geobacter* sp. strain IAE dihaloeliminates 1,1,2-trichloroethane and 1,2-dichloroethane. Environ Sci Technol 56:3430-3440.

13. Wang H, Jin H, Wang J, Wang X, Li X, Yan J, Yang Y. 2025. *Dehalogenimonas* strain W from estuarine sediments dechlorinates 1,2-dichloroethane under elevated salinity. Environ Sci Technol 59:779-790.

14. Frank JA, Reich CI, Sharma S, Weisbaum JS, Wilson BA, Olsen GJ. 2008. Critical evaluation of two primers commonly used for amplification of bacterial 16S rRNA genes. Appl Environ Microbiol 74:2461-2470.

15. Yang Y, Higgins SA, Yan J, Simsir B, Chourey K, Iyer R, Hettich RL, Baldwin B, Ogles DM, Löffler FE. 2017. Grape pomace compost harbors organohalide-respiring *Dehalogenimonas* species with novel reductive dehalogenase genes. ISME J 11:2767-2780.

16. Siddaramappa S, Challacombe JF, Delano SF, Green LD, Daligault H, Bruce D, Detter C, Tapia R, Han S, Goodwin L, Han J, Woyke T, Pitluck S, Pennacchio L, Nolan M, Land M, Chang YJ, Kyrpides NC, Ovchinnikova G, Hauser L, Lapidus A, Yan J, Bowman KS, da Costa MS, Rainey FA, Moe WM. 2012. Complete genome sequence of *Dehalogenimonas lykanthroporepellens* type strain (BL-DC-9^T^) and comparison to "*Dehalococcoides*" strains. Stand Genomic Sci 6:251-264.

17. Schumacher W, Holliger C, Zehnder AJB, Hagen WR. 1997. Redox chemistry of cobalamin and iron-sulfur cofactors in the tetrachloroethene reductase of *Dehalobacter restrictus*. FEBS Letters 409:421-425.

18. Wang H, Wang X, Huang S, Yang S, Liao H, Wang X, Jin H, Wang J, Li X, Yan J, Yang Y. 2025. Complete genome sequence of "*Candidatus* Dehalogenimonas loeffleri" strain W, a highly salt-tolerant chlorinated alkane-dechlorinating bacterium isolated from estuarine sediments. Microbiol Resour Announc 14:e0031924.

19. Magnuson JK, Romine MF, Burris DR, Kingsley MT. 2000. Trichloroethene reductive dehalogenase from *Dehalococcoides ethenogenes* sequence of *tceA* and substrate range characterization. Appl Environ Microbiol 66:5141-5147.

20. Krajmalnik-Brown R, Hölscher T, Thomson IN, Saunders FM, Ritalahti KM, Löffler FE. 2004. Genetic identification of a putative vinyl chloride reductase in *Dehalococcoides* sp. strain BAV1. Appl Environ Microbiol 70:6347-6351.

21. Tang S, Edwards EA. 2013. Identification of *Dehalobacter* reductive dehalogenases that catalyse dechlorination of chloroform, 1,1,1-trichloroethane and 1,1-dichloroethane. Philos Trans R Soc Lond B Biol Sci 368:20120318.

22. Padilla-Crespo E, Yan J, Swift C, Wagner DD, Chourey K, Hettich RL, Ritalahti KM, F.E. L. 2014. Identification and environmental distribution of *dcpA*, which encodes the reductive dehalogenase catalyzing the dichloroelimination of 1,2-dichloropropane to propene in organohalide-respiring *Chloroflexi*. Appl Environ Microbiol 80:808-818.

23. Low A, Zhao S, Rogers MJ, Zemb O, Lee M, He J, Manefield M. 2019. Isolation, characterization and bioaugmentation of an acidotolerant 1,2-dichloroethane respiring *Desulfitobacterium* species from a low pH aquifer. FEMS Microbiol Ecol 95.

24. Chen G, Kara Murdoch F, Xie Y, Murdoch RW, Cui Y, Yang Y, Yan J, Key TA, Löffler FE. 2022. Dehalogenation of chlorinated ethenes to ethene by a novel isolate, “*Candidatus* Dehalogenimonas etheniformans”. Appl Environ Microbiol 88:e0044322.

25. Molenda O, Quaile AT, Edwards EA. 2016. *Dehalogenimonas* sp. strain WBC-2 genome and identification of its trans-dichloroethene reductive dehalogenase, TdrA. Appl Environ Microbiol 82:40-50.

26. Muller JA, Rosner BM, Von Abendroth G, Meshulam-Simon G, McCarty PL, Spormann AM. 2004. Molecular identification of the catabolic vinyl chloride reductase from *Dehalococcoides* sp. strain VS and its environmental distribution. Appl Environ Microbiol 70:4880-4888.

27. Parthasarathy A, Stich TA, Lohner ST, Lesnefsky A, Britt RD, Spormann AM. 2015. Biochemical and EPR-spectroscopic investigation into heterologously expressed vinyl chloride reductive dehalogenase (VcrA) from *Dehalococcoides mccartyi* strain VS. J Am Chem Soc 137:3525-3532.

28. Cheng D, He J. 2009. Isolation and characterization of "*Dehalococcoides*" sp. strain MB, which dechlorinates tetrachloroethene to *trans*-1,2-dichloroethene. Appl Environ Microbiol 75:5910-5918.

29. Sung Y, Fletcher KE, Ritalahti KM, Apkarian RP, Ramos-Hernández N, Sanford RA, Mesbah NM, Löffler FE. 2006. *Geobacter lovleyi* sp. nov. strain SZ, a novel metal-reducing and tetrachloroethene-dechlorinating bacterium. Appl Environ Microbiol 72:2775-2782.

30. Gerritse J, Renard V, Pedro Gomes TM, Lawson PA, Collins MD, Gottschal JC. 1996. *Desulfitobacterium* sp. strain PCE1, an anaerobic bacterium that can grow by reductive dechlorination of tetrachloroethene or ortho-chlorinated phenols. Arch Microbiol 165:132-140.

31. Maillard J, Regeard C, Holliger C. 2005. Isolation and characterization of Tn-Dha1, a transposon containing the tetrachloroethene reductive dehalogenase of *Desulfitobacterium hafniense* strain TCE1. Environ Microbiol 7:107-117.

32. Miller E, Wohlfarth G, Diekert G. 1998. Purification and characterization of the tetrachloroethene reductive dehalogenase of strain PCE-S. Arch Microbiol 169:497-502.

33. Nonaka H, Keresztes G, Shinoda Y, Ikenaga Y, Abe M, Naito K, Inatomi K, Furukawa K, Inui M, Yukawa H. 2006. Complete genome sequence of the dehalorespiring bacterium *Desulfitobacterium hafniense* Y51 and comparison with *Dehalococcoides ethenogenes* 195. J Bacteriol 188:2262-2274.

34. Tsukagoshi N, Ezaki S, Uenaka T. 2006. Isolation and transcriptional analysis of novel tetrachloroethene reductive dehalogenase gene from *Desulfitobacterium* sp. strain KBC1. Appl Environ Microbiol 69:543-553.

35. Goris T, Schubert T, Gadkari J, Wubet T, Tarkka M, Buscot F, Adrian L, Diekert G. 2014. Insights into organohalide respiration and the versatile catabolism of *Sulfurospirillum multivorans* gained from comparative genomics and physiological studies. Environ Microbiol 16:3562-3580.

36. Neumann A, Wohlfarth G, Diekert G. 1996. Purification and characterization of tetrachloroethene reductive dehalogenase from *Dehalospirillum multivorans**. J Biol Chem 271:16515-16519.

37. Buttet GF, Holliger C, Maillard J. 2013. Functional genotyping of *Sulfurospirillum* spp. in mixed cultures allowed the identification of a new tetrachloroethene reductive dehalogenase. Appl Environ Microbiol 79:6941-6947.

38. Zhao J-S, Manno D, Beaulieu C, Paquet L, Hawari JA-A. 2005. *Shewanella sediminis* sp. nov., a novel Na^+^-requiring and hexahydro-1,3,5-trinitro-1,3,5-triazine-degrading bacterium from marine sediment. Int J Syst Evol Microbiol 55 Pt 4:1511-1520.

39. Wang S, Zhang W, Yang KL, He J. 2014. Isolation and characterization of a novel *Dehalobacter* species strain TCP1 that reductively dechlorinates 2,4,6-trichlorophenol. Biodegradation 25:313-323.

40. Tront JM, Amos BK, Löffler FE, Saunders FM. 2006. Activity of *Desulfitobacterium* sp. strain Viet1 demonstrates bioavailability of 2,4-dichlorophenol previously sequestered by the aquatic plant Lemna minor. Environ Sci Technol 40:529-535.

41. Thibodeau J, Gauthier A, Duguay M, Villemur R, Lepine F, Juteau P, Beaudet R. 2004. Purification, cloning, and sequencing of a 3,5-dichlorophenol reductive dehalogenase from *Desulfitobacterium frappieri* PCP-1. Appl Environ Microbiol 70:4532-4537.

42. Loffler FE, Sanford RA, Tiedje JM. 1996. Initial characterization of a reductive dehalogenase from *Desulfitobacterium chlororespirans* Co23. Appl Environ Microbiol 62:3809-3813.

43. Adrian L, Rahnenführer J, Gobom J, Hölscher T. 2007. Identification of a chlorobenzene reductive dehalogenase in *Dehalococcoides* sp. strain CBDB1. Appl Environ Microbiol 73:7717-7724.

44. Kube M, Beck A, Zinder SH, Kuhl H, Reinhardt R, Adrian L. 2005. Genome sequence of the chlorinated compound-respiring bacterium *Dehalococcoides* species strain CBDB1. Nat Biotechnol 23:1269-1273.

45. Wang S, Chng KR, Wilm A, Zhao S, Yang K-L, Nagarajan N, He J. 2014. Genomic characterization of three unique *Dehalococcoides* that respire on persistent polychlorinated biphenyls. PNAS 111:12103-12108.

46. Seidel K, Kühnert J, Adrian L. 2018. The complexome of *Dehalococcoides mccartyi* reveals its organohalide respiration-complex is modular. Front Microbiol 9:1130.

47. Wagner A, Cooper M, Ferdi S, Seifert J, Adrian L. 2012. Growth of *Dehalococcoides mccartyi* strain CBDB1 by reductive dehalogenation of brominated benzenes to benzene. Environ Sci Technol 46:8960-8968.

48. DeWeerd KA, Mandelco L, Tanner RS, Woese CR, Suflita JM. 1990. *Desulfomonile tiedjei* gen. nov. and sp. nov., a novel anaerobic, dehalogenating, sulfate-reducing bacterium. Arch Microbiol 154:23-30.

49. Kim S-H, Harzman C, Davis JK, Hutcheson R, Broderick JB, Marsh TL, Tiedje JM. 2012. Genome sequence of *Desulfitobacterium hafniense* DCB-2, a Gram-positive anaerobe capable of dehalogenation and metal reduction. BMC Microbiol 12:21.

50. Yang Y, Yan J, Li X, Lv Y, Cui Y, Kara-Murdoch F, Chen G, Löffler FE. 2020. Genome sequence of "*Candidatus* Dehalogenimonas etheniformans" strain GP, a vinyl chloride-respiring anaerobe. Microbiol Resour Announc 9.

51. Key TA, Richmond DP, Bowman KS, Cho YJ, Chun J, da Costa MS, Rainey FA, Moe WM. 2016. Genome sequence of the organohalide-respiring *Dehalogenimonas alkenigignens* type strain (IP3-3^T^). Stand Genomic Sci 11:44.

52. Kruse T, Goris T, Maillard J, Woyke T, Lechner U, de Vos W, Smidt H. 2017. Comparative genomics of the genus *Desulfitobacterium*. FEMS Microbiol Ecol 93:fix135.

53. Goris T, Hornung B, Kruse T, Reinhold A, Westermann M, Schaap PJ, Smidt H, Diekert G. 2015. Draft genome sequence and characterization of *Desulfitobacterium hafniense* PCE-S. Stand Genomic Sci 10:15.

54. Finneran KT, Forbush HM, VanPraagh CV, Lovley DR. 2002. *Desulfitobacterium metallireducens* sp. nov., an anaerobic bacterium that couples growth to the reduction of metals and humic acids as well as chlorinated compounds. Int J Syst Evol Microbiol 52:1929-1935.

55. Kruse T, van de Pas BA, Atteia A, Krab K, Hagen WR, Goodwin L, Chain P, Boeren S, Maphosa F, Schraa G, de Vos WM, van der Oost J, Smidt H, Stams AJ. 2015. Genomic, proteomic, and biochemical analysis of the organohalide respiratory pathway in *Desulfitobacterium dehalogenans*. J Bacteriol 197:893-904.
