# Supplementary figures and images for: Substrate-driven microbial specialization and cooperative dechlorination of chlorinated pollutants in estuarine ecosystems
Source: Appl Environ Microbiol. 2026 Jun 2;92(7):e00235-26. doi: 10.1128/aem.00235-26 (PMC13390484; doi:10.1128/aem.00235-26)

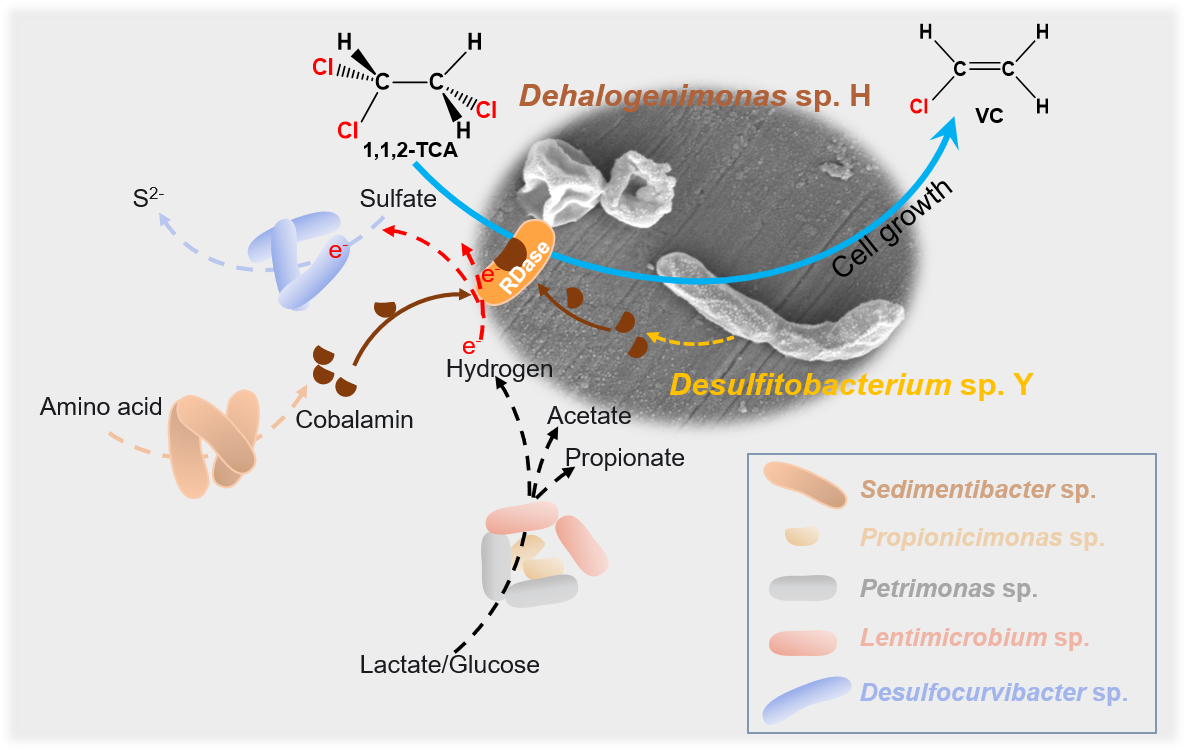

Supplement: Graphical abstract — Visual depiction of the study highlights. [file aem.00235-26-s0002.tif]
